# Back to an ice-free future: Early Cretaceous seasonal cycles of sea surface temperature and glacier ice

**DOI:** 10.1126/sciadv.adr9417

**Published:** 2025-05-02

**Authors:** Songlin He, Tianyang Wang, Robert A. Spicer, Alex Farnsworth, Andreas Mulch, Mike Widdowson, Qinghai Zhang, Fulong Cai, Paul J. Valdes, Chao Wang, Hasina Nirina Randrianaly, Jing Xie, Lin Ding

**Affiliations:** ^1^ State Key Laboratory of Tibetan Plateau Earth System, Environment and Resources (TPESER), Institute of Tibetan Plateau Research, Chinese Academy of Sciences, Beijing 100101, China.; ^2^ University of Chinese Academy of Sciences, Beijing 100049, China.; ^3^ School of Environment, Earth and Ecosystem Sciences, The Open University, Milton Keynes MK7 6AA, UK.; ^4^ School of Geographical Sciences, University of Bristol, Bristol BS8 1SS, UK.; ^5^ Senckenberg Biodiversity and Climate Research Centre, Frankfurt 60325, Germany.; ^6^ Goethe University Frankfurt, Institute of Geosciences, Frankfurt 60438, Germany.; ^7^ School of Environment Science, University of Hull, Hull Hu6 7RX, UK.; ^8^ Sedimentary Basin Evolution and Conservation, Faculty of Sciences BP 906, University of Antananarivo, Antananarivo 101, Madagascar.

## Abstract

Global ice losses will likely continue with ongoing climate warming, culminating in an almost ice-free planet analogous to that which persisted throughout much of the Cretaceous. Despite extensive research, Early Cretaceous cryosphere responses to temperature and atmospheric *P*_CO_2__ fluctuations over short, human, timescales remain uncertain. Here, we show rapid late Valanginian (~133 million years ago) seasonal fluctuations in sea surface temperature (SST) and δ^18^O mainly driven by atmospheric *P*_CO_2__. Two distinctive features emerge: large seasonal variability of up to 15.9° ± 4.9°C in Southern Hemisphere mid-latitudes, comparable to that found today, a positive sea surface δ^18^O value related to evaporation (expressed as salinity increases), and the existence of polar ice. Model-predicted patterns of SST change match with high statistical confidence those derived from clumped isotopes in well-preserved oyster fossils from Madagascar and display consistent warm/cold seasonality. Given its relative coolness in a Cretaceous context, the late Valanginian is a valuable analog for Earth’s future climate.

## Teaser

Future climate is signposted in Early Cretaceous oyster records of ocean seasonal temperatures.

## INTRODUCTION

**Fig. 1.**
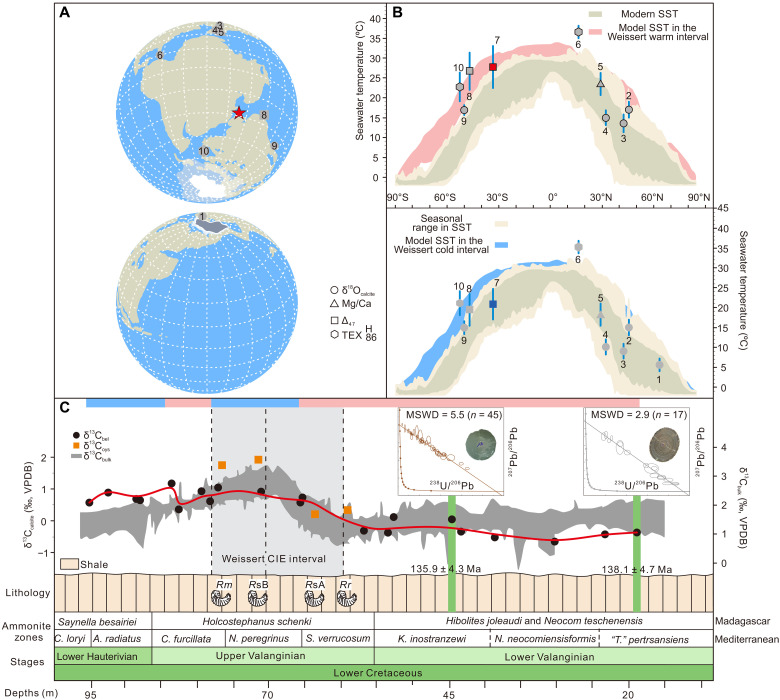
Early Cretaceous Valanginian global palaeogeography and location and age of sampling areas. (**A**) Valanginian palaeogeographic reconstruction, polar orthographic projection illustrating the actual size of (~4 million km^2^) south polar icecap, and sampling location (Mahajanga Basin, red five-pointed star). G. Projector (https://www.giss.nasa.gov/tools/gprojector/) was used to produce this polar orthographic projection (more details can be found in fig. S1). (**B**) Global SSTs for Weissert warm and cold intevals during the Valanginian. Circles show mean δ^18^O-derived seawater temperatures, squares show mean Δ_47_-derived seawater temperatures, triangles show mean belemnite Mg/Ca-derived seawater temperatures, and hexagons show mean TEX_86_-derived seawater temperatures. (**C**) Mahajanga Basin Valanginian stratigraphic column showing oyster fossil (fig. S2) sampling beds and calcite U-Pb age constraints. A previous composite global δ^13^C curve from bulk carbonate records (δ^13^C_bulk_), along with the δ^13^C variation curve from belemnite rostra (δ^13^C_bel_) indicated by a red line (*15*). Also included are measured δ^13^C values from oyster shells (δ^13^C_oys_). These are displayed adjacent to the colored bar representing Δ_47_-derived seawater temperature reconstructions, where pink signifies warming intervals and blue denotes cooling intervals. *Rm*, *Rastellum* (*Rastellum*) sp. cf. *R. macroptera* (J. de C. Sowerby, 1825); *Rr*, *Rastellum* (*Arctostrea*) *rectangularis* (Roemer, 1839); *R*sA, *Rastellum* sp. A; *R*sB, *Rastellum* sp. B.

Throughout the Cretaceous period, spanning approximately 145 to 66 million years, Earth’s climate experienced notable fluctuations (*1*–*4*). These have been determined meticulously in the marine realm through the analysis of stable (carbon and oxygen) isotopes from the carbonate shells of ancient organisms like belemnites, foraminifers, and bivalves, which serve as invaluable archives providing a rich tapestry of climatic trends, periodic variations, and transient events that have shaped Earth’s climate over millions of years [e.g., (*5*, *6*)]. The Weissert Event, which lasted approximately 1 million years (Fig. 1) and which occurred in the late Valanginian [~133 million years ago (Ma)], stands out as particularly intriguing (*7*, *8*). Paleoclimatic reconstructions from this time indicate a global cooling interval, characterized by the lowest Cretaceous sea level (*9*, *10*), modern seawater oxygen isotope composition (δ^18^O_sw_) (*11*), the presence of polar ice (*8*, *12*), and low atmospheric *P*_CO_2__ (*13*). While these macroscale climate phenomena provide valuable insights into the late Valanginian climate during the Weissert interval (*14*, *15*), the understanding of shorter-term variations, ranging from sub-annual to decadal scales, remains limited. Yet, these shorter timescales are crucial as they are commensurate with human timescales, offering insights into the seasonal dynamics of climate and their implications for long-term trends. In particular, seasonal variability plays a fundamental role in driving the hydrological cycle and climate dynamics (*4*). By bridging the gap between long-term climatic shifts and more short-term fluctuations, a deeper understanding of seasonal variability during the late Valanginian period offers invaluable insights into the broader mechanisms driving the Earth’s climate system.

Over the past few decades, reconstructions based on trace elements and δ^18^O values in shallow water corals and mollusk bivalve shells have revealed insights into climatic conditions during the ontogeny of these organisms [e.g., *16*–*19*]. Furthermore, periodically formed growth lines together with high calcification rates allow for seasonal to interannual environmental reconstructions with a precise geochronological constraint (*20*, *21*). However, δ^18^O thermometers in biogenic calcite are controlled by assumed δ^18^O_sw_, which remains poorly constrained in Phanerozoic oceans and is variable due to changes in evaporation (and thus salinity) as well as continental ice volumes (*5*, *11*). These nonthermal influences introduce uncertainties in the use of δ^18^O as a thermometer for the Cretaceous, during which seawater carbonate chemistry appears to have been highly variable (*22*). Previous studies have also suggested that Mg/Ca ratios act as proxies for seasonal upwelling based on a link between Mg/Ca fluctuations in bivalve shells and variable water temperatures (*20*, *23*–*25*). Unfortunately, like stable oxygen isotopes, trace elements can be influenced by physiological and environmental factors. Therefore, independent estimates of Tethyan Ocean temperatures during the Valanginian play a crucial role in understanding forcing and feedbacks in a dynamic climate system.

Here, we explore the evolution of seasonality in Cretaceous Tethyan Ocean sea surface temperatures (SSTs) by applying the clumped isotope (Δ_47_) thermometer, which has been defined as the excess abundance of doubly substituted carbonate isotopologs containing both ^13^C and ^18^O and is directly related to the formation temperature of biogenic calcite [e.g., (*26*–*29*)]. This method allows for accurate temperature reconstructions independent of seawater chemistry (*18*, *30*–*36*) but is subject, in some cases, to biological effects (*32*, *33*). We also analyze intrashell variations in the carbon (δ^13^C) and oxygen (δ^18^O) isotopic composition of the oyster genus *Rastellum* from Valanginian localities (~15 m stratigraphically above a horizon U-Pb dated at 135.9 ± 4.3 Ma) (*15*) in the Mahajanga Basin of the northwest of Madagascar, at a paleolatitude of ~32° to 35°S (Getech Plc.; Fig. 1A and fig. S1A). Oysters always inhabit marine intertidal environments well above the thermocline and have been the primary source of proxy data on SSTs (*17*, *34*). Our paleoclimate estimates, together with the climate modeling evidence and previous reconstructions, are used to explore absolute SST and δ^18^O_sw_ seasonality fluctuations, as well as the potential for the existence of polar ice in an Early Cretaceous greenhouse world.

## RESULTS

### Stable isotope and trace element results

**Fig. 2.**
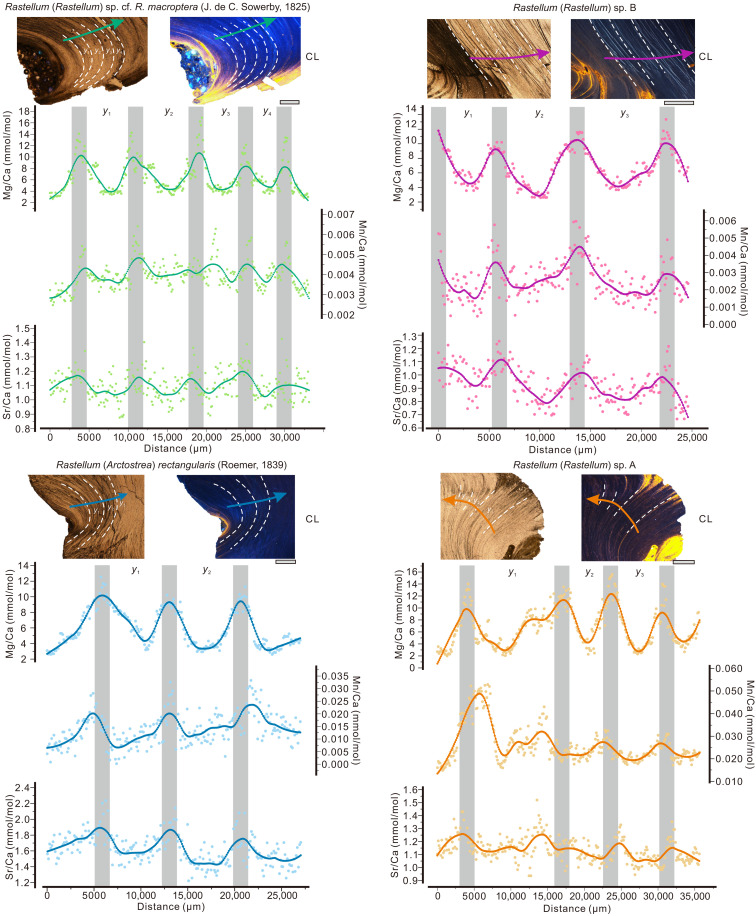
Polished cross sections and overview of element/Ca ratios of the Valanginian oysters *Rastellum*. Cathodoluminescence (CL) microscopy analyses show no diagenetic alteration in the sampled areas. Arrows indicate sample direction. White dashed lines indicate annual growth bands in the oyster shells. The locally weighted regression (LOESS) smoothing curves of Mg/Ca, Mn/Ca, and Sr/Ca and approximations of the Mg/Ca profiles by the age model routine (gray bands) plotted against modeled shell age.

**Fig. 3.**
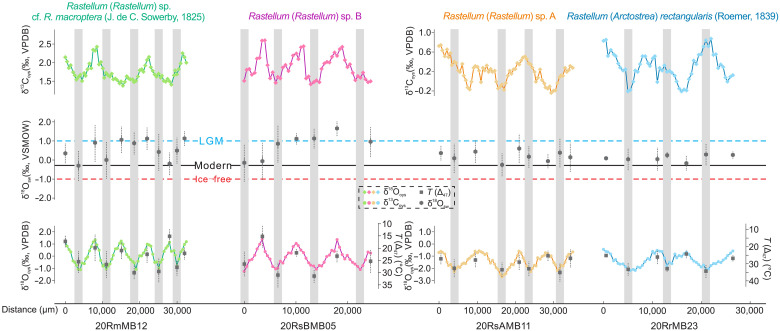
Geochemical results of genus *Rastellum* reveal the seasonal patterns of the oysters during their ontogenetic stage. δ^13^C and δ^18^O are in per mil relative to Vienna Peedee belemnite (VPDB); Δ_47_-derived temperatures are *T*(Δ_47_) and in °C; δ^18^O_sw_ values are in per mil relative to SMOW. Error bars of Δ_47_ and δ^18^O_sw_ represent 68% (solid lines) and 95% (dashed lines) confidence intervals based on multiple replicate analyses. Distance was measured from the first sampling location. Gray bands correspond to geochemical results of *Rastellum* shells during the warm months (3-warm) of the year. Horizontal blue and red dotted lines are conventional 1‰ and −1‰ Vienna standard mean ocean water (VSMOW) values for mean ocean δ^18^O during Last Glacial Maximum (LGM) and ice-free time intervals, respectively. Black horizontal solid line is the conventional −0.28‰ VSMOW value for the estimated global average δ^18^O_sw_ for the modern ocean. Refer to the main text for a description of the calculation of δ^18^O_sw_ values from Δ_47_-based temperatures and δ^18^O values of the belemnite calcite.

The stable isotope data (δ^13^C and δ^18^O) and trace element data of four individual *Rastellum* samples *Rastellum* sp. cf. *R. macroptera* (J. de C. Sowerby, 1825) (*Rm*); *Rastellum* (*Arctostrea*) *rectangularis* (Roemer, 1839) (*Rr*); *Rastellum* sp. A (*R*sA); and *Rastellum* sp. B (*R*sB) are summarized in Figs. 2 and 3 and tables S1 and S2. A global cooling of seawater during the Weissert carbon isotope excursion (CIE) event has been inferred from multiproxy ocean temperature records (*8*, *14*, *15*). This cooling phase is referred to as the Weissert cold interval (WCI). Conversely, the warming period outside of this cooling event is known as the Weissert warm interval (WWI; Fig. 1C).

Within the WWI, δ^13^C values from *R*sA and *Rr* exhibit periodic fluctuations around a stable baseline, with relatively small variations ranging from −0.39 to 0.85 per mil (‰). The δ^18^O values of *R*sA and *Rr* display three and four complete cyclic variations, respectively, reflecting the effects of seasonal changes in incident solar radiation at nonequatorial latitudes. The Mg/Ca, Mn/Ca, and Sr/Ca ratios of two oyster shells show fluctuations generally in phase with the δ^18^O values. These variations exhibit distinct banding patterns, indicating well-recorded seasonal patterns and the absence of diagenetic alteration, which is further supported by cathodoluminescence analyses. For *RsA*, the correlation between δ^18^O and the different element/Ca ratios are generally low.

During the WCI, both *R*sB and *Rm* display periodic fluctuations in δ^13^C values, oscillating around 1.70‰, with a range spanning from 1.38‰ to 2.58‰. In contrast, larger intershell disparities are evident in δ^18^O values when comparing oysters from the WWI. The *R*sB shell records exhibit three complete δ^18^O cycles, characterized by amplitudes ranging from −1.25‰ to 1.37‰, akin to the amplitude observed in the measured δ^18^O of *Rm*. Moreover, fluctuations in Mg/Ca, Mn/Ca, and Sr/Ca ratios of the two oyster shells generally align with the δ^18^O values (Figs. 2 and 3). It is important to acknowledge that the disparate sampling techniques used for element/Ca (laser) and δ^18^O (hand-held drill) analyses may affect the robustness of the correlation.

### Reconstruction of intra-annual growth models

Using the intra-annual growth rate and the ambient water temperature model proposed by Judd *et al.* (*37*), we quantified the daily ontogenetic growth trends of four oyster specimens over 3- to 5-year periods (fig. S3 and the Materials and Methods). The model results revealed consistent seasonal growth patterns and climatic fluctuations across all specimens: the warm season, defined as the three warmest months (3-warm), occurred in January, February, and March (southern hemisphere summer), coinciding with the fastest shell growth rates, reaching up to 63.4 μm/day (fig. S3). Conversely, the cold season, defined as the three coldest months (3-cold), occurred in July, August, and September (southern hemisphere winter), corresponding to the slowest shell growth rates, with a minimum of just 2.3 μm/day (fig. S3).

However, environmental water temperatures derived from the oxygen isotope thermometer indicated that the amplitude of seasonal variability in *Rs*A and *Rr* specimens from the WWI interval was notably lower compared to *RsB* and *Rm* specimens from the WCI interval. The reconstruction of intra-annual growth models facilitates direct comparisons between clumped isotopic data, modeling outputs, and modern climatic datasets within a unified reference framework.

### Clumped isotope results

Clumped isotope (Δ_47_) compositions of oyster calcite shells are summarized in the Supplementary Materials. Each sample included at least three replicates of the powder samples (table S2). The Δ_47_ values for the Valanginian Weissert warm and cold intervals range from 0.647‰ to 0.717‰. The 95% confidence level (95% CL) ranges from 0.002‰ to 0.018‰ (table S3). The lowest and highest Δ_47_ values correspond to the warm season (3-warm: July-August-September, Southern Hemisphere Summer) in the WWI recorded by *R*sA and cold season (3-cold: January-February-March, Southern Hemisphere Winter) in the WCI recorded by *Rm*, respectively. The Δ_47_ values exhibited no correlation with δ^13^C and moderate correlation with δ^18^O (fig. S4).

### Seasonal SSTs and δ^18^O_sw_ results

The sections characterized by ^18^O-enriched and lower Mg/Ca ratio segments exhibit Δ_47_-derived average temperatures of 20.1°, 19.7°, 27.3°, and 25.7°C and average δ^18^O_sw_ values of 0.94‰, 0.73‰, 0.39‰, and 0.08‰ in four studied oyster specimens. These temperatures are considered representative of SSTs during cold seasons in the Valanginian Weissert interval. Conversely, in transects displaying low δ^18^O values and high Mg/Ca ratios, the calcite in oyster shells indicate notably higher average SSTs of 28.6°, 27.9°, 33.6°, and 33.5°C and more negative δ^18^O_sw_ values of 0.74‰, 0.25‰, 0.10‰, and 0.25‰ corresponding to warmer seasons. The maximum seasonal transitions, observed prominently in specimens *R*sB, result in Δ_47_-derived SSTs varying between 15.1° ± 4.6°C and 31.0° ± 5.4°C, with a range of 15.9° ± 4.9°C (95% CL), and in δ^18^O_sw_ values varying between −0.04‰ ± 1.14‰ and 0.84‰ ± 1.02‰ (95% CL). We also calculated the mean annual range of temperature (MART), defined here as the difference between the warm season (3-warm) temperature and the cold season (3-cold) temperature. The reconstructed average MART for specimens *Rm* (8.8°C) and *R*sB (9.2°C) is higher than that observed in specimens *R*sA (6.1°C) and *Rr* (7.9°C) during the WWI. It is important to note that the geochemical results obtained from the shells studied here do not include data from the juvenile stages of oyster development. This omission is due to concerns about potential diagenetic alteration and isotopic biases that may occur during this early growth phase (*36*).

## DISCUSSION

### Relationship between clumped isotopes and temperature and possible “vital effects”

It is crucial to assess whether the megafossils accurately preserve primary environmental information. While our assessment of burial alteration, based on the petrography and high-resolution trace element concentrations, suggests that the Δ_47_ values recorded by oysters are primary, there is a possibility that solid-state reordering may have altered the clumped isotopic composition of these fossil shells [e.g., (*38*–*40*)]. Previous studies on the thermal evolution history and burial history in the Mahajanga Basin provide evidence to exclude the effect of solid-state reordering (*15*, *41*). In addition, differences in the Δ_47_-temperature calibration could influence absolute reconstructed temperatures. To address this, calibration equations with a wider temperature range and more comprehensive sample types have been used to calculate comparison temperatures [e.g., (*31*, *42*–*45*)], and using the Δ_47_-temperature calibration of Anderson *et al.* (*46*) for this study yields nominally cooler temperatures (fig. S5).

**Fig. 4.**
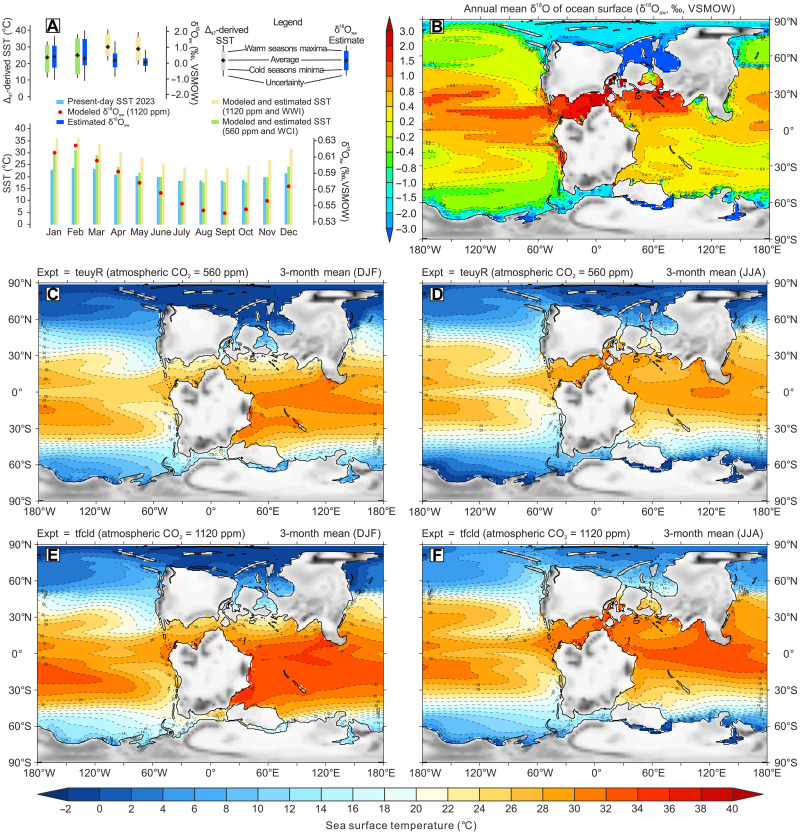
Early Cretaceous (Valanginian) global climate seasonal characteristics reconstruction. (**A**) Seasonal variations in SST and δ^18^O_sw_ values reconstructed from clumped isotopes and model simulations and compared with present-day seawater observations (monthly, derived from The National Oceanic and Atmospheric Administration Physical Sciences Laboratory, http://psl.noaa.gov), note that Δ_47_-derived SST reconstructions only distinguish between warm and cold seasons. (**B**) Valanginian global sea surface δ^18^O_sw_ value simulations. (**C** and **D**) Valanginian SSTs simulations for the atmospheric *P*_CO_2__ = 560 ppm scenario. (**E** and **F**) Valanginian SST simulations for the atmospheric *P*_CO_2__ = 1120 ppm scenario. DJF, December, January, February; JJA, June, July, August. Paleogeography from Getech Plc.

When applying the calibration of Kele *et al.* (*47*) between Δ_47_ and temperature, updated by Bernasconi *et al.* (*48*), directly to the measured megafossil Δ_47_-derived temperatures, there is an average decrease of 5.6°C (table S2). Under this scenario, the average Δ_47_-derived temperatures are 24.0° and 29.6°C, and the δ^18^O_sw_ values are −0.63‰ and −0.22‰ in the Weissert cold and warm intervals, respectively (table S2). In the studied Mahajanga Basin, under open marine conditions, there is enrichment in ^18^O compared to the ice-free global ocean average. However, the δ^18^O_sw_ values obtained are approximately similar to the reconstructed [e.g., (*11*, *15*)] and modeled δ^18^O_sw_ values in the Early Cretaceous mid-latitude Tethyan Ocean (Fig. 4B). At this stage, we followed the suggestion of Huyghe *et al.* (*36*), that paleoclimate studies of clumped isotopes in bivalves should consider using more precise calibrations such as those proposed by Anderson *et al.* (*46*) or Peral *et al.* (*49*).

Another potential reason that would bias clumped isotope results toward warmer or colder temperatures is the manifestation of kinetic isotope effects in bivalves, resulting in distinct clumped isotope disequilibrium. Calibration studies on modern and fossil organisms, including cephalopods (*50*), corals (*51*, *52*), echinoids (*32*), and brachiopods (*53*–*55*), have indicated discrepancies between Δ_47_-derived temperatures and ambient temperatures. These studies have also highlighted Δ_47_ offsets compared to other biogenic and abiogenic carbonates, possibly attributed to processes (“vital effects”) within these organisms. In addition, minor clumped isotopic variations have been observed in microstructures of bivalve species like *Megapitaria aurantiaca* (Sowerby) and in juvenile sections of oyster species such as *Magallana gigas* (Thunberg) (*33*, *36*). While exceptions exist [refer to (*33*, *56*)], in almost all cases, the inferred disequilibrium leads to higher Δ_47_ values, corresponding to cooler temperatures. Therefore, uncertainty remains regarding whether “vital effect”-induced disequilibrium precipitation occurs in Cretaceous oyster shells. Further investigations using the dual Δ_47_-Δ_48_ approach could potentially elucidate this uncertainty [e.g., (*52*, *54*, *55*)].

### Seasonal geochemical signals, age calibration, and shell growth

*Rastellum* sp., an ancient Tethyan nonrudist genus that thrived in shallow marine environments (*57*), exhibits growth patterns reminiscent of modern shallow-water oysters such as *Crassostrea* sp. The element/Ca ratios (including Sr, Mg, and Mn) in *Rastellum* fossil shells align with those found in contemporary marine oysters and display comparable seasonal patterns [e.g., (*19*, *58*)]. While Mg/Ca ratios have been proposed as thermometers for foraminifers (*59*), their application to other marine carbonate organisms such as belemnites and bivalves with broader habitat ranges still requires exploration (*40*, *60*, *61*). This knowledge gap arises because of potential biases introduced by factors such as pH levels, salinity, seawater Mg/Ca ratios, and vital effects (*19*, *62*). However, recent studies examining both modern and fossil oyster shells throughout their life spans indicate that Mg/Ca seasonality serves as a highly reliable tool for age determination, achieving a 97% success rate (*34*, *63*). Consistency between Mg/Ca and seasonality is further substantiated by comparisons with inter-annual δ^18^O_oys_ profiles in all four *Rastellum* species (refer to the Supplementary Materials for details). The oyster growth model captures effectively seasonal variations in Mg/Ca profiles across the oysters’ life span by incorporating growth rate and seasonality sinusoids (Fig. 2), affirming previous findings (*20*, *43*) that Mg/Ca profiles in *Rastellum* offer a dependable basis for age determination.

In individual specimens of *Rastellum*, the δ^18^O_oys_ results show periodic fluctuations along the growth direction, which are correlated and synchronized with Mg/Ca profiles. As supported by optical and chemical data (Fig. 2), these variations are manifest as clear banding, which reflects pristine climate signals and attests to a well-recorded seasonal pattern, including temperature and salinity. The cyclic δ^18^O_oys_ variations are characterized by a sine function and trace the seasonal variation of solar radiation at the mid-latitudes of the Southern Hemisphere (*17*). High δ^18^O_oys_ values (cold months) of fossils *Rm* and *R*sB are similar to reported δ^18^O values of Valanginian belemnite calcite collected from the low- to mid-latitudes of both hemispheres (*14*, *64*) and likely indicate deeper seawater signals than those of oysters (*5*).

Although modern climatology typically defines seasonality based on 30-year averages (*65*), very few paleontological records from geological history provide continuous 30-year time series. Fossil records from individual accretionary organisms often represent less than 10 years of growth, making paleo-seasonal parameters derived from such records exceptionally rare. Nevertheless, these data are critically important for understanding seasonal variability during key periods and in key regions of Earth’s past (*65*, *66*). Most current studies rely on high-resolution seasonal data from one or a few individual specimens (≤5 years) to reconstruct temperature seasonality in ancient and/or modern oceans [e.g., (*17*, *33*)] or even past monsoon systems [e.g., (*66*, *67*)].

In contrast to the δ^18^O values, δ^13^C values are controlled by multiple factors. The relevance of carbon isotopes in well-preserved carbonate fossils as an indicator of dissolved inorganic carbon (DIC) has been questioned because of taxonomic differences (vital effects) (*58*). The average values of two lower Valanginian specimens exhibit good agreement, thus suggesting that the vital effects in *Rastellum* sp. are not expressed as notable isotope biases and fluctuations. A positive CIE of 1.5‰ has been observed between the WWI (*R*sA and *Rr*) and WCI (*R*sB and *Rm*) Valanginian specimens (Fig. 1C). This CIE may be correlated to the Weissert event (*7*, *68*), which represents a global carbon cycle fluctuation and has been verified in marine and terrestrial settings (*68*, *69*). However, this hypothesis cannot be further tested because of the absence of the high-resolution δ^13^C stratigraphy in Madagascar. Although all studied shells show the generally small variation in δ^13^C values, the utility of carbon isotope ratios for tracking seasonal seawater DIC fluctuation remains uncertain because this proxy is controlled by environmental change, such as late spring phytoplankton blooms (*58*) and/or metabolic processes.

### Early Cretaceous SST fluctuations and latitudinal temperature gradient

Our late Valanginian mid-latitude temperature estimates from ~15° to 35°C, mean of ~27°C, are slightly different from previous SST mean annual temperature reconstructions of 22.7°C within the WWI at a higher latitude (~54°S) based on TEX_86_ (*8*). This difference is consistent with a shift of ~12° in latitude under a shallow latitudinal temperature gradient in the late Valanginian Southern Hemisphere. However, at this stage, our temperatures are representative of only a narrow mid-latitude band between 32° to 35°S and ~54°S because of the limited latitude and temperature ranges covered by analyzed samples and are insufficient to calculate reliably the overall gradient. For comparison, a latitudinal temperature gradient of 0.32°C per degree of latitude has been estimated by recent Δ_47_ and TEX_86_ data in the Northern Hemisphere (*11*, *70*, *71*). The occurrence of such shallow temperature gradients compared with those of today requires a climate mechanism in a greenhouse world with warm tropics and cool, but not cold, polar regions (*11*). Moreover, paleoclimate reconstructions suggest that the Late Jurassic–Early Cretaceous interval is characterized by warm global seawater temperatures and high atmospheric *P*_CO_2__ and support shallow latitudinal thermal gradients (*70*, *72*). A lower seasonality in our two specimens during the WWI at mid-latitude is also compatible with a shallow equator-to-pole temperature gradient.

Seawater temperature seasonality data from the Early Cretaceous is limited, particularly for regions in low- to mid-latitudes. However, when compared with Late Cretaceous seasonality data, our findings indicate that reconstructed MARTs are lower than those at higher latitudes, although the annual SSTs are higher in the Late Cretaceous (*18*). This observation is worth comparing with the present-day Earth situation, in which seasonal temperature ranges are generally much higher in Northern Hemisphere mid- to high-latitudes (30° to 50°N) than in Southern Hemisphere low- to mid-latitudes (10° to 30°S) (Fig. 1B). Such reconstructions of seasonality based on geological evidence and paleoclimate models suggest that the average MART in the greenhouse world (e.g., Cretaceous ×4 preindustrial atmospheric *P*_CO_2__ levels) was similar to modern rather than more equable (*18*, *73*, *74*).

### Model-proxy comparisons of the Early Cretaceous climate

Coupled ocean-atmosphere numerical general circulation climate modeling has provided valuable insights into coupling global sea level, atmospheric *P*_CO_2__, polar ice sheet dynamics, and δ^18^O of seawater over geological timescales, particularly during greenhouse periods [e.g., (*75*–*77*)], including for the Valanginian (*78*, *79*). Leaf stomata results suggest that the range of available Valanginian *P*_CO_2__ reconstructions is ~500 to 1700 parts per million (ppm) (*80*). Our Δ_47_-derived Valanginian annual and seasonal SSTs in the mid-latitude Southern Hemisphere exhibit close correspondence with simulated early Cretaceous paleoclimate results under varying boundary conditions (Fig. 4). The associated atmospheric *P*_CO_2__ levels are estimated as nearly ×4 preindustrial *P*_CO_2__ levels (940 to 1480 ppm) for the WWI and nearly ×2 preindustrial *P*_CO_2__ levels (550 to 840 ppm) for the WCI, respectively (*8*). This alignment between SST reconstructions and different simulated *P*_CO_2__ levels suggests a global mechanism, with fluctuations in atmospheric *P*_CO_2__driving temperature variations in the Cretaceous greenhouse world and δ^13^C excursions in marine and terrestrial environments (*13*, *68*).

According to estimates derived from a paleosol barometer in the low- to mid-latitudes of the Northern Hemisphere, atmospheric *P*_CO_2__ ranged from approximately 241 to 530 ppm during the interval spanning the early Valanginian to Barremian (*81*). Cavalheiro *et al.* (*8*) recently proposed a reduction in *P*_CO_2__ from around 1180 to 680 ppm between the warm pre-CIE and cold CIE periods, respectively, suggesting that this decrease in atmospheric *P*_CO_2__, coupled with global cooling, may have contributed to the potential build-up of polar ice. The observation that our reconstructed cold seasonal SSTs at 32° to 35°S resemble present-day conditions and coldest 3-month means in the ×2 preindustrial *P*_CO_2__ simulation, which has been regarded as a *P*_CO_2__ threshold for the onset of Antarctic glaciation for the Early Cretaceous (*76*), becomes challenging to explain in the context of a supposedly ice-free world (Fig. 4). In the mid-latitude regions of the Southern Hemisphere during the Valanginian, model simulations indicate that the MART was ~11°C at 560 ppm *P*_CO_2__ levels and ~12°C at 1120 ppm (table S4). These findings align well with our Δ_47_-derived MARTs (table S4), suggesting that MART shows limited sensitivity to variations in atmospheric CO_2_ levels. This implies that for every doubling of CO_2_ concentration, the MART increases by only ~1° to 2°C.

### Early Cretaceous δ^18^O_sw_ reconstructions and the implications for the seasonal SSTs and Glacier ice

Oceanic δ^18^O_sw_ has evolved throughout the Phanerozoic in concert with major tectonic, climatic, and biologic events, and the values reflect a global dynamic balance between water-rock reactions over geological timescales (*22*). The average of our calculated δ^18^O_sw_ [Vienna standard mean ocean water (VSMOW)] value is 0.1‰ based on the Daëron *et al.* (*31*) equation for the Valanginian and is more positive than δ^18^O_sw_ values of assumed ice-free climate conditions (*11*), but it is close to the mean value for the modern oceans (−0.28‰ VSMOW) (fig. S6) (*78*). According to Price *et al.* (*11*), there are four potential explanations for the difference between calculated, assumed, and modern δ^18^O_sw_ values, including carbonate diagenesis, kinetic isotope (vital) effects, uncertainties in the appropriate Δ_47_-temperature calibration, and the chemical evolution of seawater. We have already addressed the impact of the first three explanations, leaving the fourth, which we shall next discuss.

Changes in δ^18^O_sw_ values can be related to changes in seawater chemistry, including salinity and seawater pH [e.g., (*82*–*84*)]. High rates of evaporation result in higher salinities and high δ^18^O_sw_ values. The oyster fossils we examined originate from an open marine environment, characterized by the presence of a fully marine fauna including ammonites and belemnites (*15*, *41*). Moreover, unusually high salinities are not consistent with our model simulations (fig. S7) and seem unlikely to have existed in the studied area. Alternatively, δ^18^O values of biogenic carbonate such as oyster shells are influenced by changes in seawater pH, which is mainly controlled by fluctuations in *P*_CO_2_._ Consequently, δ^18^O_sw_ values reconstructed from δ^18^O values of biogenic carbonate are indirectly influenced by changes in seawater pH (*11*). An excursion of ~1.6‰ in reconstructed δ^18^O_sw_ (VSMOW) values (from an assumed ice-free −1‰ to our calculated 0.6‰ in the late Valanginian) would require ~1.6 pH units to achieve this effect. Accordingly, we consider such a magnitude of change as unlikely.

In addition to the possibilities noted above, late Valanginian δ^18^O_sw_ values that are comparable with modern characteristics may imply some storage of ^18^O-depleted water in reservoirs outside the ocean (e.g., ice sheet expansion in Antarctica) (*11*). Numerous indicators of cold environments including dropstones, tillite deposits, ice rafted debris, and glendonites, have been recognized in Valanginian deposits in both polar region (e.g., Australian, Canadian, Spitsbergen, and Siberian basins) (*85*–*88*) and suggest therefore the existence of modest-sized ice sheets during the Cretaceous (*8*, *89*–*92*). While modest-sized continental ice sheets from the Early Cretaceous have been hypothesized (*11*, *91*), this volume (approximately 16.5 × 10^6^ km^3^), which is roughly half of the current Antarctic ice sheet, likely would not result in δ^18^O_sw_ values exceeding modern levels. Consequently, any Early Cretaceous ice sheets would have had an isotopically lighter composition of oxygen isotope (δ^18^O_ice_), likely below −80‰ (fig. S8). If the δ^18^O_ice_ value were less negative, achieving δ^18^O_sw_ values similar to those of today would be even more challenging, as even larger continental ice volumes would be necessary to account for the difference.

It is worth noting that the paleoclimatic significance of glendonites remains controversial (*93*, *94*), but clumped isotope thermometry on glendonites can provide estimates on the upper limit of 2 to 5 (±3)°C for temperatures at the sediment/water interface (*94*). Significant increases in regional and global mean salinity from the WWI to the WCI have been observed in our paleoclimate model results (fig. S6), suggesting that freshwater may have been stored as ice on land as a consequence of global cooling. In addition, estimates of Cretaceous eustatic sea level changes suggested that notable fluctuations of ~65 m occurred within the Valanginian, which is considered a magnitude characteristic for glacio-eustasy (*10*).

Given the reconstructed and modeled positive δ^18^O_sw_ values, together with above-freezing annual mean SST in the polar regions that seem to have existed during the WWI (fig. S9), it is important to consider the nature of the drivers of the seasonal continental ice that can cause δ^18^O_sw_ values with modern characteristics. Intuitively, in a warm world such as existed in the Cretaceous, the presence of ice is likely to alternate between the Arctic and Antarctica on a seasonal basis, but in climate models, ice sometimes persists throughout the year in the polar regions. Of course, these assessments have their limitations, particularly associated with (lack of) resolution and incomplete characterization of local and regional processes in large-scale climate models.

The seasonal seawater temperatures and geochemical results reported here suggest that the WCI may have been characterized by transient, high-latitude glaciations triggered by shifts in radiative forcing and/or reductions in atmospheric CO_2_ levels. The “coolhouse” conditions at mid-latitude regions of the Southern Hemisphere during the WCI bear similarities to those of today’s climate, particularly in terms of the comparably low atmospheric CO_2_ concentrations, SSTs during colder seasons, δ^18^O_sw_ values, and the global proportion of land. Conversely, the WWI appears to represent an intermediate climatic state, influenced by orbital forcing that periodically nudged conditions toward either an ice-free state or one with seasonal ice coverage. This analysis builds upon two key observations. First, the Δ_47_-derived SST seasonality during the WCI aligns with modeled results that assume ×2 preindustrial *P*_CO_2__ levels (560 ppm), a finding corroborated by global stomatal and geochemical proxy data (*80*). Second, consistently positive δ^18^O_sw_ values and rapidly fluctuating salinities, likely due to ice formation and/or melting in polar regions, support this conclusion.

Applying our findings to the present-day offers a key insight: Given ongoing and anticipated global warming, reductions in Arctic and Antarctic ice are expected to continue, potentially leading to completely ice-free polar regions. Despite uncertainties in these predictions, we transition from a world with perennial glacier ice to one with only seasonal ice or shifting from a predominantly white winter planet to a blue one. Using the Valanginian as a model for predicting future climate changes, biota migration, and extinction events could prove beneficial. This approach is justified not merely by its greenhouse condition, but because it exemplifies an intermediate state with variable glacier sizes and a likely scenario under escalating global warming.

## MATERIALS AND METHODS

### Chronostratigraphic framework

The age determination of all Valanginian oyster fossils relies on the analysis of strontium isotopes within a well-established chronostratigraphic framework (fig. S10). This framework has been established on the basis of calcite U-Pb ages of belemnite rostra and the Sr isotope curve, aligning with biostratigraphy in the Ankirihitra section [refer to (*15*) for detailed information]. We drilled approximately 4 mg of powder from the intermediate shell layers of the oysters. Strontium separation is achieved through an optimized and simplified dissolution scheme, with the advantage of rapid operation using a Triton Plus multicollector thermal ionization mass spectrometry at the State Key Laboratory of Lithospheric Evolution, Institute of Geology and Geophysics, Chinese Academy of Sciences, Beijing, China [IGGCAS; refer to (*95*) for detailed methods]. To ensure the stability of instruments during data collection, the international standard sample NBS987 is used, yielding an ^87^Sr/^86^Sr ratio of 0.710258 ± 0.000012 (2 SE). Numerical ages are calculated using the “look-up” table provided by McArthur *et al.* (*96*). The statistical uncertainty associated with the means of the ^87^Sr/^86^Sr values is combined with the uncertainty linked to the seawater strontium-isotope curve for accurate age determination.

### Laser ablation trace element analyses

Trace element profiles are obtained using a ESL 193-nm ArF ultraviolet excimer laser with an ANU Helex dual-volume laser ablation cell coupled to an Agilent 7900 quadrupole inductively coupled plasma mass spectrometer at the State Key Laboratory of Tibetan Plateau Earth System, Environment and Resources (TPESER) in Institute of Tibetan Plateau Research, Chinese Academy of Sciences (ITPCAS) following Wang *et al.* (*41*). Helium serves as the carrier gas during the analysis. Rather than using a line scan method, high-resolution transects are obtained in point-by-point mode to ensure discrete spot sampling without overlap. This approach results in an effective sampling resolution of 120 μm in the measurement direction.

The isotopes ^43^Ca, ^24^Mg, ^88^Sr, ^55^Mn, and ^57^Fe are used to calculate Mg/Ca, Sr/Ca, Mn/Ca, and Fe/Ca ratios. Calibration of element/Ca ratios in calcium carbonate shells is conducted using a National Institute of Standards and Technology (NIST) SRM 612 glass standard, whose accuracy for various elements is validated using a 193-nm laser. Raw data are processed using Iolite 4.0 software to set time windows for data integration, followed by calibration and data evaluation. The concentration of calcium in CaCO_3_ (40.08%) is used as a reference, with ^43^Ca serving as an internal standard. The long-term SD (2σ) for the element/Ca ratio is typically better than 7% based on analyses of the NIST SRM 612 standards.

### Intra-annual growth model and associated δ^18^O variations

We used the quantitative model of Judd *et al.* (*37*) to calculate the intra-annual growth rates and environmental water temperatures of the fossil oysters. This model is considered applicable to a wide range of accretionary biogenic skeletal materials, including bivalves, fish otoliths, corals, etc. and can be applied to long time series when incremental width measurements would otherwise be impractical or unfeasible to attain (*37*, *65*).

Samples were collected from the clean shell layer using a stationary dental drill at slow rotation speed equipped with a 75-μm-diameter tungsten carbide drill bit. Viewing with a stereomicroscope allowed us to drill holes perpendicular to the growth bands in all *Rastellum* shells, with the sample locations placed adjacent to each other, at a spacing of approximately 0.5 mm between adjacent holes (about 20 drilled holes per cm). Oxygen and carbon isotope analyses of the drilled powders were conducted at the Laboratory for Stable Isotope Geochemistry in IGGCAS. Isotopic measurements were made using a Thermo Fisher Scientific MAT 253 mass spectrometer coupled with a GasBench II preparation system. Approximately 0.3 mg of powdered calcite samples was reacted with 100% orthophosphoric acid at 75°C within the preparation unit, producing CO_2_ gas, which was then analyzed by the isotope ratio mass spectrometer. The precision of the analyses was better than ±0.03‰ for δ^13^C and ±0.05‰ for δ^18^O, as determined by replicate measurements of two international carbonate standards (NBS-19 and IAEA-603). All C─O isotope ratios are presented in per mil relative to the Vienna Peedee belemnite (VPDB) standard. The model assumes that annual temperature variations follow a sinusoidal curve, and it is used to determine the intra-annual growth rate function that most closely matches the observed δ^18^O data. Specifically, δ^18^O values, determined along the developmental trajectory of the individual organism’s shell, are used to quantify the spatial and temporal patterns of intra-annual accretion within populations.

In this study, we obtained δ^18^O-derived temperature and growth function outputs from four samples (see details in table S5). By analyzing the variations in oxygen isotope profiles (*y* axis, δ^18^O values) at different positions along the distance axis (*x* axis, distance) of the shells, we quantified the individual ontogenetic trends of fossil oysters over 3 to 5 years. This analysis provides insight into the temporal variations in growth curves as well as climate fluctuations (fig. S3).

### Clumped isotope analyses

The onset and end of each growth year are defined on the basis of maximum Mg/Ca ratios, which also show remarkable consistency to intra-annual growth model (grays bands in Figs. 3 and 4). Subsequently, the warm (3-warm) and cold (3-cold) seasons for each growth year, positioned precisely relative to the intra-annual growth model and seasonal temperature cycle, were chosen for clumped isotope analysis at the TPESER, ITPCAS. Every sampling location included at least three replicates of the powder samples, and the number of tests is shown in the Δ_47_-temperature results in Fig. 3 and table S2. For each powder sample, approximately 1 ml of phosphoric acid (ρ = ~1.93 g/cm^3^, ~104%) is used for digestion in a common acid bath at 90°C for 15 min. The generated CO_2_ samples were then purified off-line before entering a Thermo Fisher Scientific 253 Plus isotope ratios mass spectrometer to obtain δ^13^C, δ^18^O, and the raw Δ_47_ data. More details for the clumped isotope analyses are given in (*91*). We use the value of 0.088‰ to augment the Δ_47-ARF_ values derived from the 90°C acid digestion absolute reference frame (ARF) value to generate Δ_47, ARF-AC_ values based on the 25°C acid digestion ARF value. For processing the raw data files, we use Easotope (ver. 20201231) (*97*) to process the raw data. We adopt the Brand parameters for calculating Δ_47_ values, with λ set at 0.528 and isotope abundances as follows: ^13^C/^12^C_VPDB_ at 0.01118, ^18^O/^16^O_VSMOW_ at 0.0020052, and ^17^O/^16^O_VSMOW_ at 0.00038475 (*98*).

To correct for nonlinearity in the raw Δ_47_ values, we use data from heated gas and 25°C equilibrated gas (*99*) and subsequently transfer them to an absolute reference frame using the empirical transfer function along with data from 25°C equilibrated gases and 1000°C heated gas (*100*). For enhancing interlaboratory data comparability, we used the ARF values of ETH carbonate standards to establish a secondary reference frame by plotting their ARF values against those reported by Bernasconi *et al.* (*101*), yielding values of 0.2965‰ for ETH-2, 0.7012‰ for ETH-3, 0.5385‰ for ETH-4, 0.3898‰ for IAEA-C1, 0.7289‰ for IAEA-C2, and 0.6015‰ for ETH-MERCK. This linear regression enables the definition of the standard transfer function (STF), which facilitates the determination of an in-house laboratory standard (Bingzhou, BZ). Using the calibrated STF-ARF-47 values of three standards (IAEA-C1, IAEA-C2, and BZ), we convert our unknown samples from ARF-Δ_47_ values to STF-ARF-Δ_47_ values (see details in table S2), using a method similar to that described by Chang *et al.* (*102*).

### Calculation of SST and δ^18^O_sw_

The Δ_47_-derived temperatures are determined using the equation proposed by Anderson *et al.* (*46*), which encompasses a broad temperature range from of 0.5° to 1100°C and accounts for various types of carbonates. This calibration equation has been successfully used in oysters such as *Pycnodonte newberryi* (Stanton), *Pycnodonte kellumi* (Jones), and *Exogyra* sp. to reconstruct mid-Cretaceous seawater temperatures (*103*) and in the bivalve *Lucina pensylvanica* (Linnaeus) (*104*). However, when using the foraminiferal-based, multilaboratory calibration by Meinicke *et al.* (*44*) or the composite calibration by Petersen *et al.* (*19*), nominally warmer temperatures and lower δ^18^O_sw_ are obtained (table S2).

To derive the δ^18^O_sw_ results from δ^18^O and the Δ_47_-derived temperatures, the slow-growing, abiotic calcite equation developed by Kele *et al.* (*47*) is used. This equation is believed to represent the true equilibrium fractionation between calcite and water, as evidenced by Daëron *et al.* (*31*). Recent studies have also applied this equation to biological calcite, yielding δ^18^O_sw_ results within the expected range for semi-enclosed basins to open marine settings [e.g., (*91*, *105*)]. Analytical uncertainties on Δ_47_, temperature, and δ^18^O_sw_ values are displayed as 95% CLs (table S3) following the recommendations by Bajnai *et al.* (*54*) and Fernandez *et al.* (*106*).

### Paleoclimate modeling

An isotope-enabled version of the HadCM3 climate model (specifically, HadCM3BL-M2.1aD) (*107*) is used in this study. This model comprises a horizontal resolution of 3.75° × 2.5° in both longitude and latitude, with 19 hybrid levels in the atmosphere and 20 levels in the ocean on an Arakawa-grid. The MOSES 2.1 land surface scheme is used with the dynamic interactive vegetation scheme, TRIFFID [Top-Down Representation of Interactive Foliage and Flora Including Dynamics; (*108*)]. TRIFFID predicts the distribution and properties of global vegetation based on five plant functional types, namely, bare soil, C3 grass, C4 grass, needleleaf trees, and broadleaf trees.

We use the same isotope module detailed in (*109*) with some changes to the numerical scheme to improve model stability. The isotope model has been shown to represent accurately modern terrestrial (*110*) and ocean (*111*) δ^18^O distributions globally. In addition, we change the ozone scheme in the model. Valdes *et al.* (*112*) has shown that for warm climates, the tropopause rises and stratospheric ozone penetrates into the troposphere, which is unphysical. The ozone scheme is instead replaced with a three-dimensional distribution scheme coupled to the tropopause height with constant values for the troposphere (0.02 ppm), tropopause (0.2 ppm), and stratosphere (5.5 ppm). Refer to (*112*) for details.

Model boundary conditions are configured for the Early Cretaceous, specifically the Valanginian (133 to 140 Ma). The solar constant is reduced by 1.15% of preindustrial (1361 W/m^2^ to 1345.39 W/m^2^), atmospheric CO_2_ is set at 1120 and 560 ppm, which is within proxy-CO_2_ uncertainty range (*80*, *113*). We use the Getech Plc. Palaeogeography [refer to (*78*) for full details]. Other radiatively active gases, aerosols, and orbital parameters are set at preindustrial levels.

All simulations are initialized from a previous Valanginian simulation (*79*), which was spun-up without isotope tracking enabled for more than 10,000 model years. The isotope-enabled simulation is run for 500 years, with the last 100 years used to form climatological means. This is sufficient for the surface ocean isotopes to reach near equilibrium but not the deep ocean. We therefore concentrate on the surface ocean isotopic values only.

## Supplementary Materials

### The PDF file includes:

Supplementary Text Figs. S1 to S10 Legends for tables S1 to S5 Legend for data S1 References
