# Supplementary material for: Back to an ice-free future: Early Cretaceous seasonal cycles of sea surface temperature and glacier ice
Source: Sci Adv. 2025 May 2;11(18):eadr9417. doi: 10.1126/sciadv.adr9417 (PMC12047435; doi:10.1126/sciadv.adr9417)
Supplement: Supplementary file 1 — Supplementary Text Figs. S1 to S10 Legends for tables S1 to S5 Legend for data S1 References [file sciadv.adr9417_sm.pdf]

Science Advances
AAAS

Supplementary Materials for

Back to an ice-free future: Early Cretaceous seasonal cycles of sea surface temperature and glacier ice

Songlin He *et al.*

Corresponding author: Tianyang Wang, wangtianyang@itpcas.ac.cn; Lin Ding, dinglin@itpcas.ac.cn

Sci. Adv. **11**, eadr9417 (2025)
DOI: 10.1126/sciadv.adr9417

The PDF file includes:

Supplementary Text
Figs. S1 to S10
Legends for tables S1 to S5
Legend for data S1
References

Other Supplementary Material for this manuscript includes the following:

Tables S1 to S5
Data S1

1. Geologic setting

The geological structure of Madagascar (fig. S1) can be categorized into two primary units: the ancient Precambrian shield and the more recent Phanerozoic sedimentary cover (*114*). The latter is notable for its passive margin basinal successions on the western side of the island, a result of Mesozoic rifting, spreading, and the eventual separation of Madagascar from the African plate, beginning around the Permo-Triassic era and concluding in the Early Cretaceous (*115*, *116*).

The Mahajanga, and Morondava basins of the west coast passive margin, contain a series of transgressive-regressive cycles that persisted until the end of the Cretaceous (*117*). Notably, the Mahajanga Basin extends from the southern Ampasindava Peninsula to Cap St. André, and offers a detailed stratigraphic history marked by alternating continental sediment pulses and marine intervals from the Callovian to the end of the Valanginian (*118*). This sedimentary continuity from the Upper Jurassic to the Cretaceous is evident in those strata outcropping between the Betsiboka and Mahavavy rivers, where basal Cretaceous deposits, such as the Ankirihitra marls and shales, extend up to the Upper Valanginian. The transition from the Berriasian to the Valanginian is marked by the abrupt disappearance of ammonites and the emergence of abundant belemnites in the Lower Valanginian, followed by a resurgence of an ammonite fauna in the Upper Valanginian, which is accompanied by the appearance of oysters. The influence of solid-state reordering of shell structure by dysgenesis in the Mahajanga Basin has been previously addressed by Wang et al. (*15*), and indicates that this phenomenon may not significantly impact the Δ_{47}-derived temperatures of oyster shells. Given that the regional Late Cretaceous igneous activity was located far from the studied section, and that no igneous intrusions are present within the Valanginian shales (*119*), along with the fact that regional faulting ceased during the Early

Cretaceous and the burial depth did not surpass 1 km (*118*, *120*), we estimate the burial temperatures of these sediments were unlikely to have exceeded 50 ℃. Considering this maximum burial temperature estimate, it's improbable that the oyster shells experienced solid-state reordering, since calcite requires prolonged exposure to temperatures above 80–120 °C over millions of years for such reordering to take place (e.g., *121*, *122*).

2. Assessment of the Valanginian oyster fossils

Oyster shells are mainly composed of calcite, with aragonite found specifically at the muscle attachment points and the ligostracum. The ligostracum forms a thin prismatic layer that secures the ligament to the hinge (*123*). While previous research has shown no significant difference in clumped isotope values between aragonite and calcite in bivalve shells, meaning a separate calibration for each mineral or an acid fractionation factor is unnecessary (*35*), this study focuses solely on sampling the calcite portions of the shell.

Calcite shell parts exhibit two distinct structural forms: foliate layers and chalky formations (*124*). The foliate layers are made up of elongated calcite crystals stacked in a slightly offset manner, with each layer oriented in a slightly different direction from the one beneath it. This intricate arrangement contributes to the overall stability and strength of the shell. In contrast, the chalky structures form a three-dimensional network that appears to be more delicate. Unlike the well-organized foliate layers, the chalky formations seem less influenced by functional requirements and may have formed more rapidly (*125*). It is suggested that these two types of structures were deposited at different rates, with the chalky layers possibly precipitating more quickly than the

foliate layers. Recent multi-isotopic and trace element results further reinforce this view, indicating that the transient nature of chalky lenses and the absence of seasonal influence on microstructure formation make it difficult to draw reliable conclusions regarding the timing of mineralization or the environmental conditions based on bulk measurements of this microstructure (*34*). In line with de Winter et al. (*34*) recommendations, the foliated structures were sampled for chemical analyses in this study (fig. S2, D and E) to account for the strong seasonal variability recorded in the shell, which can otherwise bias environmental reconstructions.

Four well preserved oyster shells from the Ankirihitra section were analysed; this succession is known for yielding well-preserved marine fossils such as belemnites and ammonites (*15, 41*). The oyster shells were sectioned perpendicular to their primary growth axis and polished for inspection in order to evaluate the preservation quality. Each section was examined using standard optical microscopy, cathodoluminescence (CL) microscopy, and electron probe micro-analysis (EPMA), using a Zeiss Axio Imager 2 Pol, a MK-CL-5200 cold cathode luminoscope, and a JOEL JXA-8230 electron microprobe, respectively. The luminoscope was set at 250 μA current and a voltage between 13–15 KV, and images were captured with a Nikon Ds-Fl3 digital camera during a 2 second exposure time at the Resources Exploration Laboratory, China University of Geosciences, Beijing.

Additionally, high-resolution X-ray elemental intensity mapping on oyster thin sections was conducted at the State Key Laboratory of Tibetan Plateau Research Earth System, Environment and Resources (TPESER), Institute of Tibetan Plateau Research, Chinese Academy of Sciences (ITPCAS) using a 300 nA probe current and a 20 kV acceleration voltage. We selected the elemental Kα line for all elements and varied the

step size from 1–4 µm, with a dwell time of 100–200 ms per point. Mapping each grain typically required between 8 to 17 hours to complete.

The cross-sectional analysis of oyster shells from the Ankirihitra section reveals a distinct pattern of light and dark growth bands; these are particularly pronounced in the ligamental area. The bands represent annual growth cycles consisting of dark-light couplets (Fig. 2), indicative of seasonal environmental changes. The methods employed in this study (i.e., petrographic and geochemical), yield the following insights into the calcite preservation within these oyster shells: (1) Optical Inspection: Examination under a microscope shows that the oyster calcite was free from dissolution features and larger cracks; this demonstrates good preservation of the shell material; (2) Cathodoluminescence (CL) Imaging: The CL analysis resolved the oyster calcite into several types: non-luminescence (NL), slightly luminescence (SL), and cathodoluminescent (CL) (i.e., a gradational scale of cathodoluminescence; refer to Fig. 6 in *126*). Those areas that exhibit blue to non-luminescence under CL microscopy indicate that these regions retain the intrinsic CL color of calcite, and are thus indicative of well-preserved material. Conversely, the inner shell layers showing SL typically indicate the presence of Mn^{2+} cations, likely revealing diagenetic alterations in the material (*127*); (3) Trace Element Analysis: The geochemical studies determined that well-preserved regions of the shells contained low levels of Mn and Fe, less than 30 ppm and 100 ppm, respectively. These findings are consistent with high-resolution X-ray elemental intensity mapping results, which similarly indicate low Mn and Fe levels. These observations are consistent with previous research regarding the geochemistry of biogenic carbonate, where primary biominerals typically show low concentrations of elements such as Mn, but diagenetic processes can lead to their enrichment (*128*). Previous studies on early Cretaceous calcite microfossils from the Mahajanga Basin

used upper limits of 0.05 mmol/mol for Mn/Ca and 0.27 mmol/mol for Fe/Ca, as being indicative of good preservation (*41*). Such criteria are also applicable to the oyster shells analyzed in this study, and therefore provide a consistent framework for assessing fossil preservation across different calcite microfossils.

3. Sr/Ca ratios in oyster calcite

The use of Sr/Ca ratios as a paleothermometer in aragonitic corals is well-established (*129*). In corals these ratios are sensitive to temperature changes, making them useful for reconstructing past climate conditions. In contrast, Sr/Ca ratios in inorganic calcite are not thermodynamically dependent on temperature, Dodd (*130*) suggests there might be other influencing factors at play. For aragonite in bivalves, the relationship between Sr/Ca ratios and environmental factors such as temperature and salinity has also been demonstrated, though the influence of biological processes on these ratios cannot be ignored (*131*). Furthermore, the Sr/Ca ratios in molluscs are often related not only to environmental factors (e.g., diagenetic alteration) but also to physiological (vital) processes, such as metabolic activity and calcification rates, as well as the Sr/Ca ratio and Mg/Ca of the surrounding seawater (*132*).

The Sr/Ca data comparison highlights striking similarities in magnitude and variability with Sr/Ca ratios found in modern Pacific oysters (*M. gigas*), ranging from 0.7 to 1.3 mmol/mol (*19, 58*). Despite these parallels, the Sr/Ca ratios in the specimen *Rr* stand out due to their higher values and broader fluctuations, and notably, they show no substantial correlation with the temperature estimates derived from Δ_{47} data. This deviation therefore raises questions about the influence of environmental versus biological factors on these measurements. Further analysis comparing peaks in Sr/Ca

ratios during both the Weissert Warm Interval and Cool Interval reveals only a moderate alignment with seasonal variations predicted by Mg/Ca ratios and δ^{18}O values (Figs. 2 and 3). This suggests that while these proxies can align, external factors may alter or mask the climate signals that they might otherwise indicate. Accordingly, this ongoing ambiguity (i.e., whether variations in Sr/Ca ratios are primarily influenced by habitat conditions or are a result of biological processes), emphasizes the intricate dynamics between environmental proxies and the elemental composition of calcitic bivalve shells. Our findings further highlight necessity for careful interpretation when utilizing Sr/Ca ratios in paleoclimate studies and we recognize the importance of accounting for both biological influences and environmental factors.

4. Mn/Ca ratios in oyster calcite

In modern shallow-water bivalves from marine and freshwater environments, seasonal variations in Mn/Ca ratios correlate with changes in dissolved and particulate manganese levels in the water, as well as with primary production and biological factors such as growth rates (*133*). Such variations highlight the need to fully understand the manganese dynamics of a specific shallow-water environment before interpreting Mn/Ca ratios from biogenic carbonates intrinsic therein. In more dynamic coastal areas such as the Mahajanga Basin, as compared to the open marine environment, both seawater and pore waters exhibit more accentuated seasonal enrichments in particulate manganese and dissolved manganese (Mn_{diss}). These enrichments are influenced by riverine inputs and the release of manganese through pore water from sediment (e.g., *134*). Furthermore, there appears to be a strong linkage between primary production and the Mn cycle within tidal basins, with tidal creeks therein showing significantly

higher concentrations of Mn_{diss} compared to those in the water column of the more open backbarrier areas (*135*). Additionally, tidal variations in Mn concentrations in seawater occur and are mediated, and affected by, the discharge of Mn-rich pore waters during low tide, largely driven by hydraulic gradients (*136*). This intricate interplay of geological and biological factors underscores the complexity of using Mn/Ca ratios as a proxy in environmental and paleoceanographic studies, and necessitates a detailed understanding of local conditions.

To summarize, the Mn/Ca ratios serve as a useful tool for assessing the preservation state of calcite fossils, and offers a semi-quantitative proxy (*41*, *91*, *128*, *137*). However, it is important to recognize that a single, global Mn/Ca threshold for identifying altered calcite in fossils is not universally obtainable or applicable. This is because potential manganese enrichments can vary significantly depending on diagenetic alterations, which are influenced by the specific fossil groups and geological settings. Thus, while Mn/Ca ratios are valuable, they should be used in conjunction with other screening techniques to obtain a more comprehensive understanding of the fossil's condition and the extent of any post-depositional changes.

5. figures S1 to S10

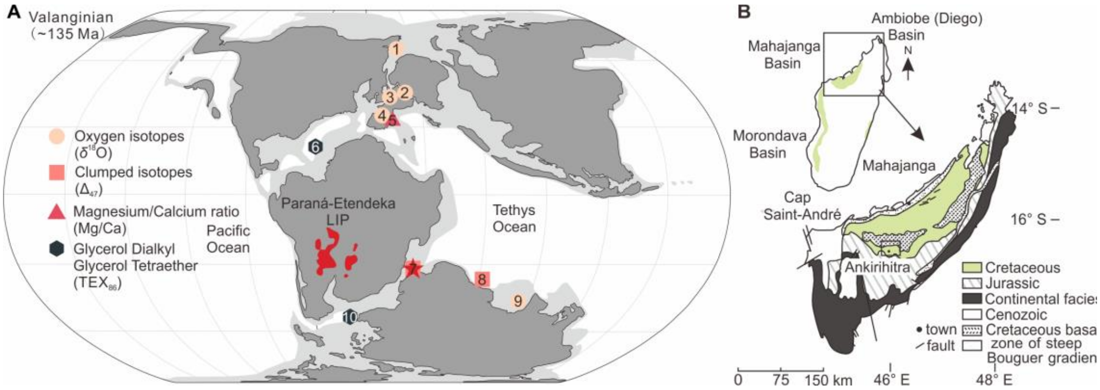

fig. S1. Geologic map of the study region. (**A**) The Getech Plc. Palaeogeography show the location of the available Early Cretaceous (Valanginian) temperature estimates including TEX_{86}, $\delta^{18}O$, Δ_{47}, and belemnite Mg/Ca ratio from worldwide sections. (1) Arctic Svalbard (*85*); (2) Lower Saxony Basin (*64*); (3) Yorkshire (*138*); (4) Vocontian Basin and the Provence Platform (*12*, *139*); (5) Vocontian Basin and Caravaca (*140*); (6) DSDP Site 534 (*70*); (7) Mahajanga Basin (*15*, this study); (8) Gyangze Basin (*91*); (9) DSDP Site 765C (*14*); (10) ODP Site 692 (*8*). (**B**) Geological map of northwest Madagascar showing the location of the Mahajanga Basin (the black block indicates the studied section shown in Fig. 1C).

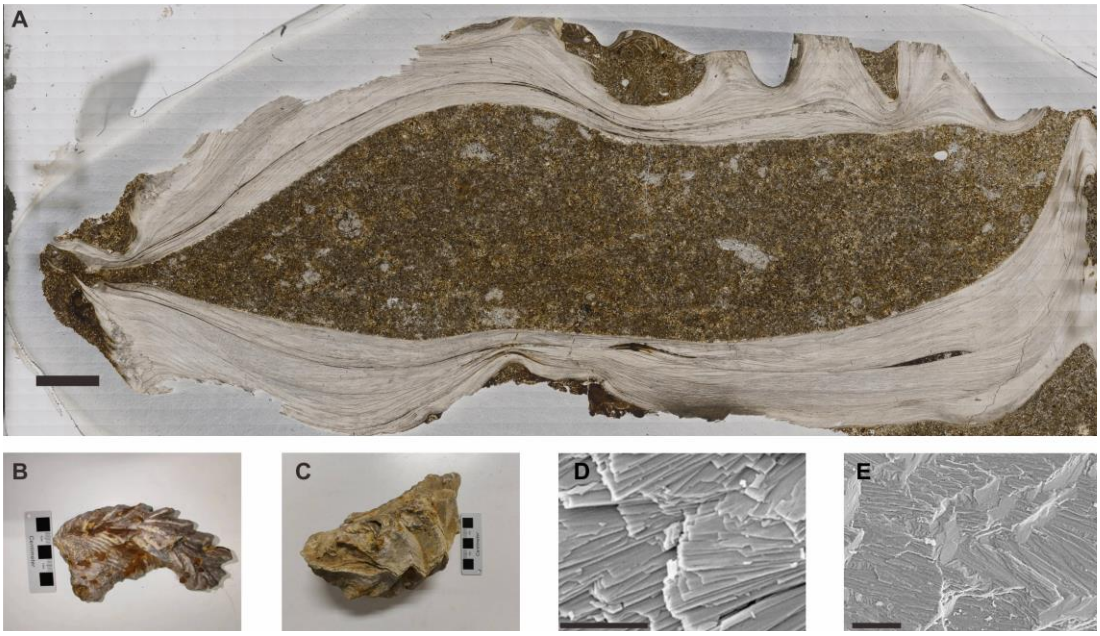

fig. S2. Photographs of *Rastellum* analyzed in this study. (**A**) Coress-sectional view of *Rastellum* sp. B. Scar bar represents 1 cm; (**B** and **C**) *Rastellum* sp. A. and *Rastellum* sp. B.; (**D** and **E**) Foliated layers in *Rastellum* (*Rastellum*) sp. cf. *R. macroptera* and *Rastellum* sp. A., respectively. Scar bar represents 10 µm.

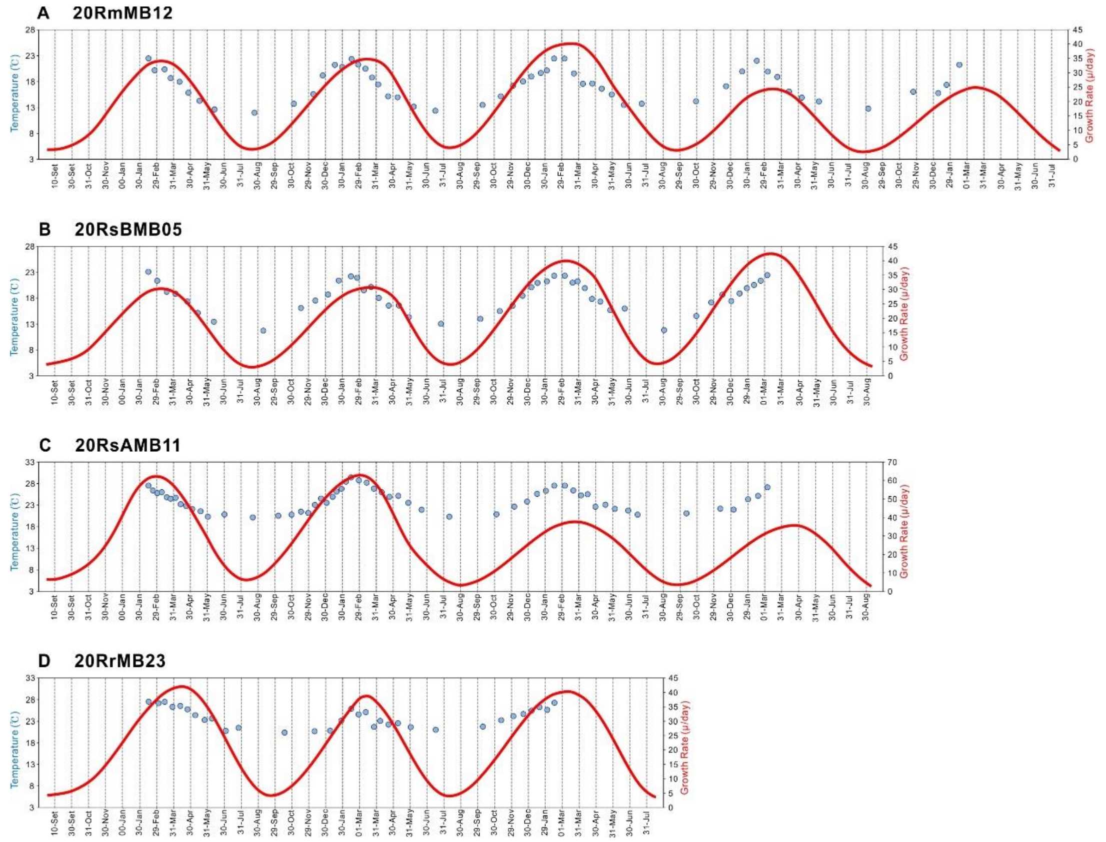

fig. S3. Intra-annual growth model and corresponding oxygen-derived ambient water temperatures of fossil oyster shells based on oxygen isotope profiles. The results show the daily growth rate changes and monthly environmental temperature fluctuations over 3–5 years. Specifically, (**A**) sample 20RmMB12 exhibits a 5-year cycle; (**B** and **C**) samples 20RsBMB05 and 20RsAMB11 display 4-year cyclical oscillations; and (**D**) sample 20RrMB23 shows a 3-year cycle. The model is based on Judd et al. (*37*), with raw data and Matlab code (data S1) provided in the supplementary materials.

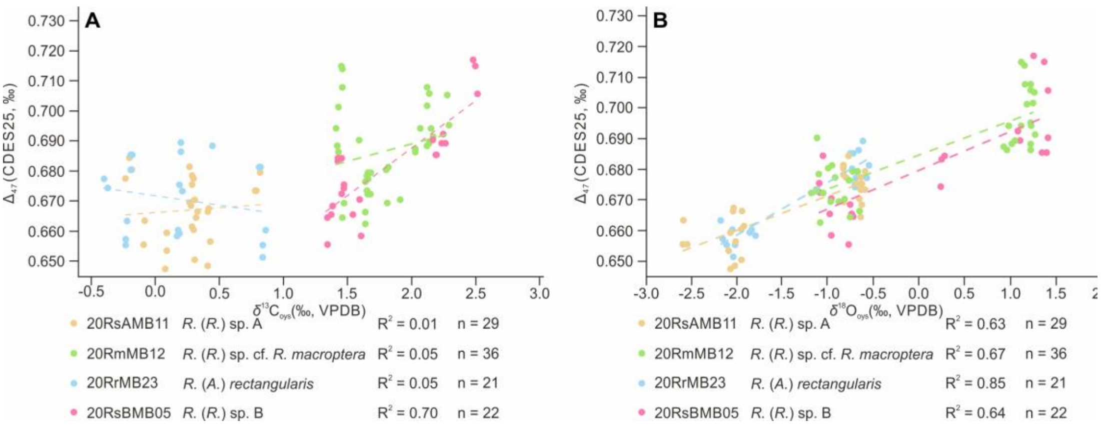

fig. S4. Shell Δ_{47}-δ^{13}C (A) and Δ_{47}-δ^{18}O (B) plots for all studied *Rastellum*. There are no Δ_{47}-δ^{13}C correlations found in specimens of *R.* sp. A., *R. rectangularis*, and *R.* sp. cf. *R. macroptera.* A significant positive correlation between Δ_{47} and δ^{13}C is evident in specimen *R.* sp. B., which we interpret as a pristine signal reflecting changes in environment, mainly via seasonal fluctuations, rather than alteration.

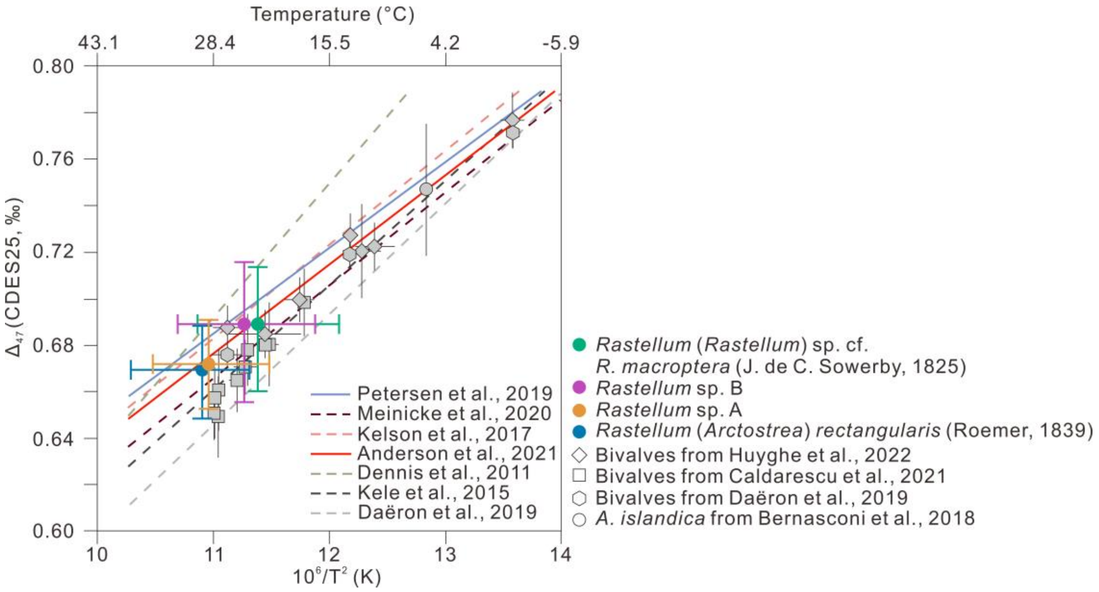

fig. S5. Comparison of various Δ_{47}-temperature calibrations. Clumped isotope data from four oyster fossils in this study (colored circles) and the calculated linear regression (colored solid and dashed lines) is compared with published Δ_{47} values from bivalves (*31*, *33*, *36*, *48*) and calibrations lines (*31*, *42–44*, *46*, *47*, *100*). The travertine calibration of Kele et al. (*47*) has been recalculated by Bernasconi et al. (*48*). Colored circles are mean estimates. Growth temperatures are calculated by Δ_{47} values and the equation of Anderson et al. (*46*). Error bars in x and y directions represent the Δ_{47} and temperature ranges and uncertainty with 95% confidence, as presented in table S3.

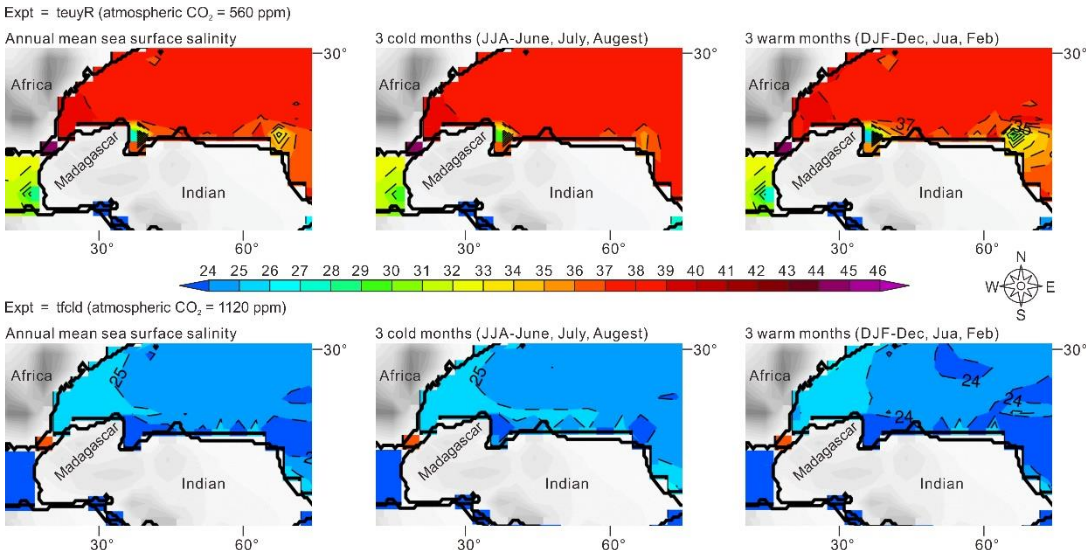

fig. S6. Regional mean sea surface salinity conditions at different short-time scales. Annual and three warm and cold months salinity in the southern part of the Tethyan Ocean at 560 and 1120 ppm CO_2.

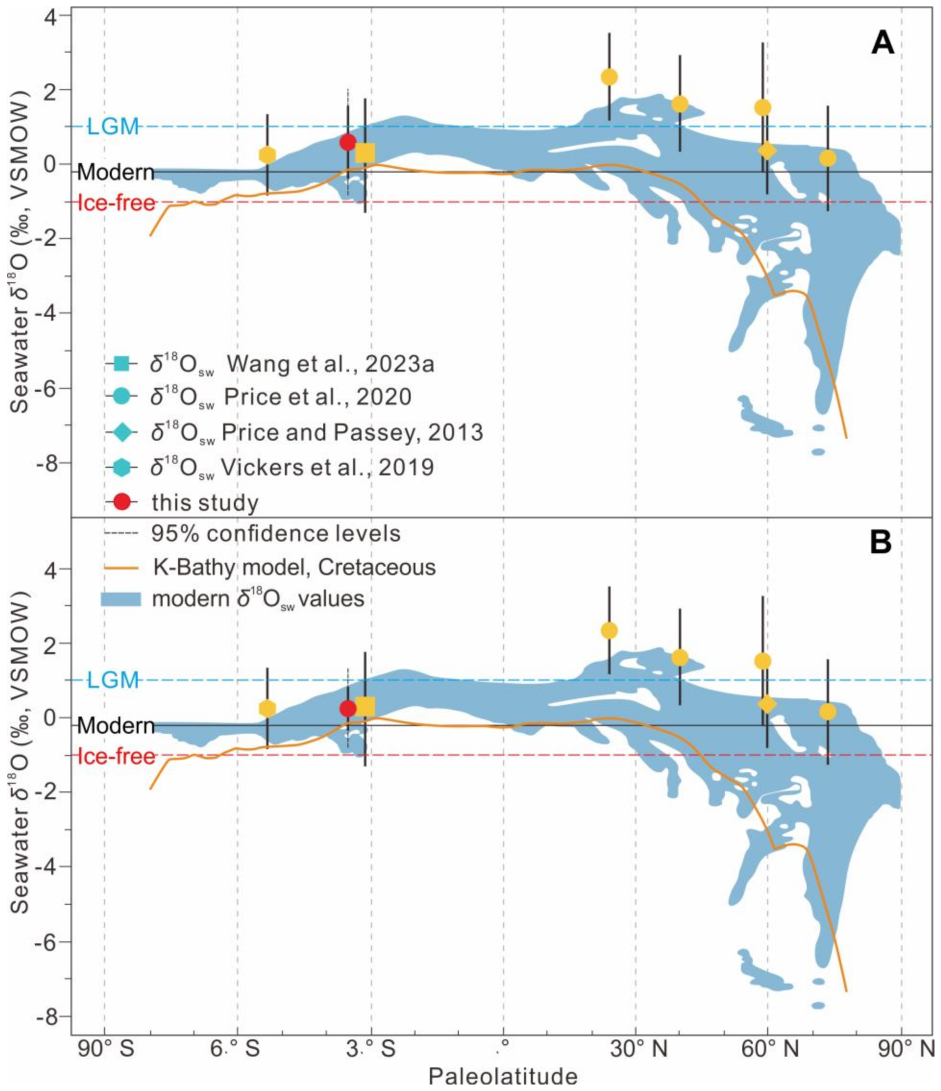

fig. S7. Valanginian meridional sea surface water oxygen isotope gradient in the Weissert cold (A) and warm (B) intervals. $\delta^{18}O_{sw}$ (‰, VSMOW) calculated using the equation of Kele et al. (*47*) (see table S2 for different results from other equations) with additional Valanginian data derived from Price and Passey (*71*), Vickers et al. (*72*), Price et al. (*11*), Wang et al. (*91*). Red circles are mean estimates and uncertainties are 95% confidence levels. The $\delta^{18}O_{sw}$ values of 1.0‰, −0.28‰, and −1.0‰ VSMOW are widely regarded as the mean seawater oxygen isotope composition for the Last Glacial Maximum (LGM), modern, and ice-free conditions (*81*).

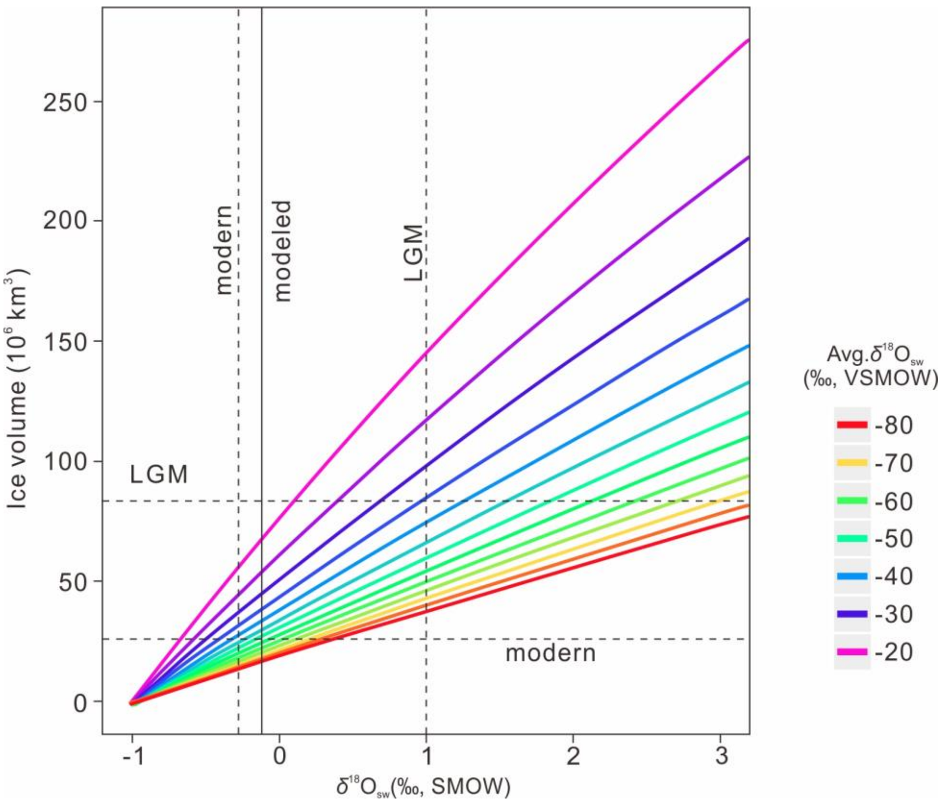

fig. S8. Inferred ice volumes (x axis) for different measured $\delta^{18}O_{sw}$ values (y axis) using different assumptions for the mean oxygen isotopic composition of Early Cretaceous glacial ice (colored lines) (modified from ref. *141*). Horizontal dashed lines indicate inferred ice volumes for the modern and LGM, vertical dashed lines indicate modern and LGM $\delta^{18}O_{sw}$ (*81*). Horizontal and vertical solid lines indicate reconstructed and modeled Early Cretaceous ice volumes and $\delta^{18}O_{sw}$ values (*91*).

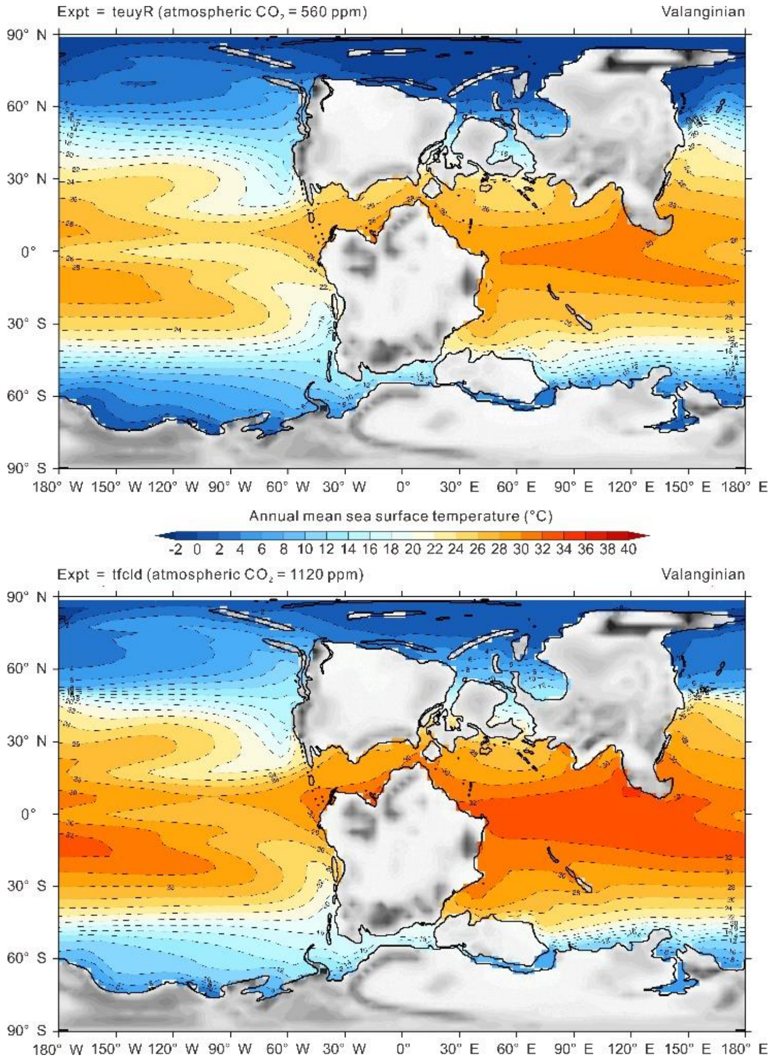

fig. S9. Global mean annual sea surface temperature for Valanginian stage at ×2 and ×4 pre-industrial atmospheric CO_2 concentrations (560 and 1120 ppm).

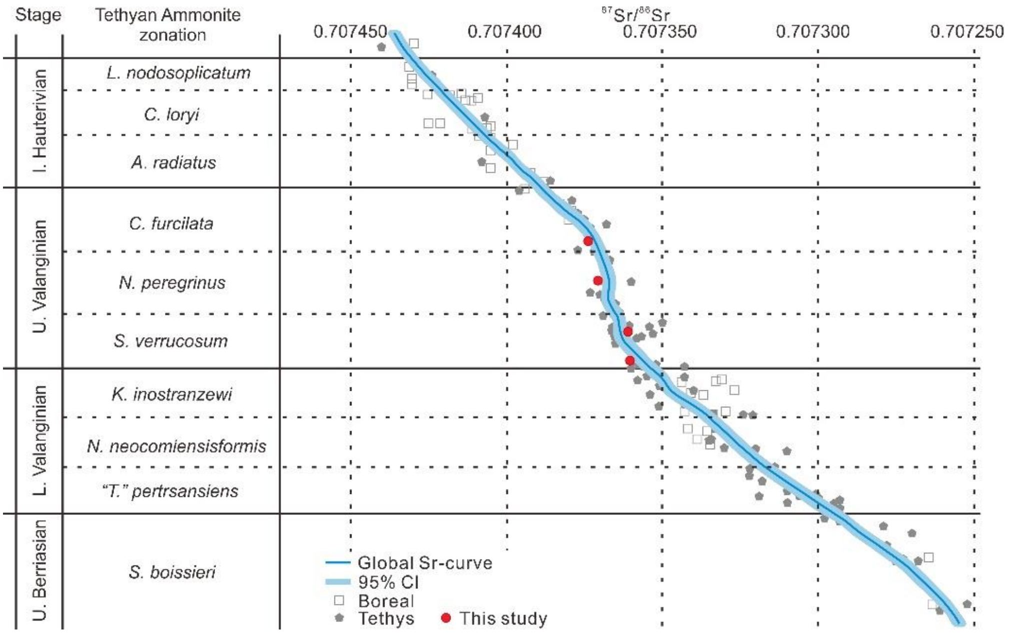

fig. S10. Global Sr-isotopes from belemnite rostra and oyster. Curves modeled with LOWESS 6 function (*12*, *96*). Tethyan and Boreal $^{87}Sr/^{86}Sr$ ratios are derived from Mutterlose et al. (*142*) and references therein.

6. Legends for tables S1 to S5

table S1. Compilation of stable isotopes and trace elements from four oysters.

table S2. Summary of the Δ_{47}, $T(\Delta_{47})$, and $\delta^{18}O_{sw}$ in this study.

table S3. Errors of the Δ_{47}, $T(\Delta_{47})$, and $\delta^{18}O_{sw}$ in this study.

table S4. Modeled and modern climatic results.

table S5. Raw data for intra-annual growth model and $\delta^{18}O$ variations.

7. Legends for data S1

data S1. Matlab code for generating the intra-annual growth model of fossil oyster shells based on oxygen isotope profiles.

REFERENCES AND NOTES

1. P. A. Wilson, R. D. Norris, Warm tropical ocean surface and global anoxia during the mid-Cretaceous period. *Nature* **412**, 425–429 (2001).

2. B. Huber, R. D. Norris, K. G. MacLeod, Deep-sea paleotemperature record of extreme warmth during the Cretaceous. *Geology* **30**, 123–126 (2002).

3. C. L. O'Brien, S. A. Robinson, R. D. Pancost, J. S. Sinninghe Damsté, S. Schouten, D. J. Lunt, H. Alsenz, A. Boremann, C. Bottini, S. C. Brassell, A. Farnsworth, A. Forster, B. T. Huber, G. N. Inglis, H. C. Jenkyns, C. Linnert, K. Littler, P. Markwick, A. McAnena, J. Mutterlose, B. D. A. Naafs, W. Püttmann, A. Sluijs, N. A. G. M. van Helmond, J. Vellekoop, T. Wagner, N. E. Wrobel, Cretaceous sea-surface temperature evolution: Constraints from TEX_{86} and planktonic foraminiferal oxygen isotopes. *Earth Sci. Rev.* **172**, 224–247 (2017).

4. R. Spicer, P. Valdes, A. Hughes, J. Yang, T. Spicer, A. Herman, A. Farnsworth, New insights into the thermal regime and hydrodynamics of the early Late Cretaceous Arctic. *Geol. Mag.* **157**, 1729–1746 (2020).

5. J. Veizer, A. Prokoph, Temperatures and oxygen isotopic composition of Phanerozoic oceans. *Earth Sci. Rev.* **146**, 92–104 (2015).

6. E. L. Grossman, M. M. Joachimski, Ocean temperatures through the Phanerozoic reassessed. *Sci. Rep.* **12**, 8938 (2022).

7. E. Erba, A. C. Bartolini, R. L. Larson, Valanginian Weissert oceanic anoxic event. *Geology* **32**, 149–152 (2004).

8. L. Cavalheiro, T. Wagner, S. Steinig, C. Bottini, W. Dummann, O. Esegbue, G. Gambacorta, V. Giraldo-Gómez, A. Farnsworth, S. Flögel, P. Hofmann, D. J. Lunt, J. Rethemeyer, S. Torricelli, E. Erba, Impact of global cooling on Early Cretaceous high P_{CO_2} world during the Weissert Event. *Nat. Commun.* **12**, 5411 (2021).

9. B. U. Haq, Cretaceous eustasy revisited. *Glob. Planet Change* **113**, 44–58 (2014).

10. D. C. Ray, F. S. P. van Buchem, G. Baines, A. Davies, B. Gréselle, M. D. Simmons, C. Robson, The magnitude and cause of short-term eustatic Cretaceous sea-level change: A synthesis. *Earth Sci. Rev.* **197**, 102901 (2019).

11. G. D. Price, D. Bajnai, J. Fiebig, Carbonate clumped isotope evidence for latitudinal seawater temperature gradients and the oxygen isotope composition of Early Cretaceous seas. *Palaeogeogr. Palaeoclimatol. Palaeoecol.* **552**, 109777 (2020).

12. J. M. McArthur, N. M. M. Janssen, S. Reboulet, M. J. Leng, M. F. Thirlwall, B. van de Schootbrugge, Palaeotemperatures, polar ice-volume, and isotope stratigraphy (Mg/Ca, δ18O, δ13C, 87Sr/86Sr): The Early Cretaceous (Berriasian, Valanginian, Hauterivian). *Palaeogeogr. Palaeoclimatol. Palaeoecol.* **248**, 391–430 (2007).

13. T. J. Jin, K. W. Huntington, Y. X. Wen, X. Gu, A. J. Schauer, L. M. Zhang, Clumped isotope records of terrestrial temperatures during the Middle Jurassic (180–150Ma) in East China. *Palaeogeogr. Palaeoclimatol. Palaeoecol.* **637**, 112014 (2024).

14. G. Charbonnier, S. Duchamp-Alphonse, J.-F. Deconinck, T. Adatte, J. E. Spangenberg, C. Colin, K. B. Föllmi, A global palaeoclimatic reconstruction for the Valanginian based on clay mineralogical and geochemical data. *Earth Sci. Rev.* **202**, 103092 (2020).

15. T. Y. Wang, P. Yang, S. L. He, R. Hoffmann, Q. H. Zhang, A. Farnsworth, Y.-X. Feng, H. N. Randrianaly, J. Xie, Y. H. Yue, J.-X. Zhao, L. Ding, Absolute age and temperature of belemnite rostra: Constraints on the Early Cretaceous cooling event. *Glob. Planet. Change* **233**, 104353 (2024).

16. K. M. Cobb, C. D. Charles, H. Cheng, R. L. Edwards, El Niño/Southern oscillation and tropical Pacific climate during the last millennium. *Nature* **424**, 271–276 (2003).

17. T. Steuber, M. Rauch, J.-P. Masse, J. Graaf, M. Malkoč, Low-latitude seasonality of Cretaceous temperatures in warm and cold episodes. *Nature* **437**, 1341–1344 (2005).

18. N. J. de Winter, I. A. Müller, I. J. Kocken, N. Thibault, C. V. Ullmann, A. Farnsworth, D. J. Lunt, P. Claeys, M. Ziegler, Absolute seasonal temperature estimates from clumped isotopes

in bivalve shells suggest warm and variable greenhouse climate. *Commun. Earth Environ.* **2**, 121 (2021).

19. J. Petersen, J. Titschack, J. Groeneveld, A. Wehrmann, D. Hebbeln, A. Freiwald, Reef-building Pacific oysters record seasonal variations in water mass properties of tidal basins from the Central Wadden Sea (North Sea). *Palaeogeogr. Palaeoclimatol. Palaeoecol.* **577**, 110534 (2021).

20. L. Bougeois, G. Dupont-Nivet, M. de Rafélis, J. C. Tindall, J.-N. Proust, G.-J. Reichart, L. J. de Nooijer, Z. J. Guo, C. Ormukov, Asian monsoons and aridification response to Paleogene sea retreat and Neogene westerly shielding indicated by seasonality in Paratethys oysters. *Earth Planet. Sci. Lett.* **485**, 99–110 (2018).

21. H. Sadatzki, M. Alberti, D. Garbe-Schönberg, N. Andersen, P. Strey, H. Fortunato, C. Andersson, P. Schäfer, Paired Li/Ca and $\delta^{18}O$ peaks in bivalve shells from the Gulf of Panama mark seasonal coastal upwelling. *Chem. Geol.* **529**, 119295 (2019).

22. U. Ryb, J. M. Eiler, Oxygen isotope composition of the Phanerozoic ocean and a possible solution to the dolomite problem. *Proc. Natl. Acad. Sci. U.S.A.* **107**, 11245–11249 (2018).

23. V. Mouchi, M. de Rafélis, F. Lartaud, M. Fialin, E. Verrecchia, Chemical la-belling of oyster shells used for time-calibrated high-resolution Mg/Ca ratios: A tool for estimation of past seasonal temperature variations. *Palaeogeogr. Palaeoclimatol. Palaeoecol.* **373**, 66–74 (2013).

24. B. R. Schöne, P. Radermacher, Z. Zhang, D. E. Jacob, Crystal fabrics and element impurities (Sr/Ca, Mg/Ca, and Ba/Ca) in shells of Arctica islandica—Implications for paleoclimate reconstructions. *Palaeogeogr. Palaeoclimatol. Palaeoecol.* **373**, 50–59 (2013).

25. L. Bougeois, M. de Rafélis, G. J. Reichart, L. J. de Nooijer, F. Nicollin, G. Dupont-Nivet, A high resolution study of trace elements and stable isotopes in oyster shells to estimate Central Asian Middle Eocene seasonality. *Chem. Geol.* **363**, 200–212 (2014).

26. N. J. de Winter, J. Tindall, A. L. Johnson, B. Goudsmit-Harzevoort, N. Wichern, P. Kaskes, P. Claeys, F. Huygen, S. van Leeuwen, B. J. S. A. Metcalfe, P. Bakker, S. Goolaerts, F. Wesselingh, M. Ziegler, Amplified seasonality in western Europe in a warmer world. *Sci. Adv.* **10**, eadl6717 (2024).

27. P. Ghosh, J. Adkins, H. Affek, B. Balta, W. Guo, E. A. Schauble, D. Schrag, J. M. Eiler, ^{13}C–^{18}O bonds in carbonate minerals: A new kind of paleothermometer. *Geochim. Cosmochim. Acta* **70**, 1439–1456 (2006).

28. J. M. Eiler, "Clumped-isotope" geochemistry—The study of naturally-occurring, multiply-substituted isotopologues. *Earth Planet. Sci. Lett.* **262**, 309–327 (2007).

29. G. A. Henkes, B. H. Passey, E. L. Grossman, B. J. Shenton, T. E. Yancey, A. Pérez-Huerta, Temperature evolution and the oxygen isotope composition of Phanerozoic oceans from carbonate clumped isotope thermometry. *Earth Planet. Sci. Lett.* **490**, 40–50 (2018).

30. R. A. Eagle, J. M. Eiler, A. K. Tripati, J. B. Ries, P. S. Freitas, C. Hiebenthal, A. D. Wanamaker, M. Taviani, M. Elliot, S. Marenssi, K. Nakamura, P. Ramirez, K. Roy, The influence of temperature and seawater carbonate saturation state on ^{13}C–^{18}O bond ordering in bivalve mollusks. *Biogeosciences* **10**, 4591–4606 (2013).

31. M. Daëron, R. N. Drysdale, M. Peral, D. Huyghe, D. Blamart, T. B. Coplen, F. Lartaud, F. G. Zanchetta, Most Earth-surface calcites precipitate out of isotopic equilibrium. *Nat. Commun.* **10**, 429 (2019).

32. A. J. Davies, C. M. John, The clumped ($^{13}C^{18}O$) isotope composition of echinoid calcite: Further evidence for "vital effects" in the clumped isotope proxy. *Geochim. Cosmochim. Acta* **245**, 172–189 (2019).

33. D. E. Caldarescu, H. Sadatzki, C. Andersson, P. Schäfer, H. Fortunato, A. N. Meckler, Clumped isotope thermometry in bivalve shells: A tool for reconstructing seasonal upwelling. *Geochim. Cosmochim. Acta* **294**, 174–191 (2021).

34. N. J. de Winter, L. K. Dämmer, M. Falkenroth, G.-J. Reichart, S. Moretti, A. Martínez-García, N. Höchem, B. R. Schöne, K. Rodiouchkina, S. Goderis, F. Vanhaecke, S. M. van Leeuwen, M. Ziegler, Multi-isotopic and trace element evidence against different formation pathways for oyster microstructures. *Geochim. Cosmochim. Acta* **308**, 326–352 (2021).

35. N. J. de Winter, R. Witbaard, I. J. Kocken, I. A. Müller, J. J. Guo, B. Goudsmit, M. Ziegler, Temperature dependence of clumped isotopes (Δ_{47}) in aragonite. *Geophys. Res. Lett.* **49**, e2022GL099479 (2022).

36. D. Huyghe, M. Daëron, M. de Rafelis, D. Blamart, M. Sébilo, Y.-M. Paulet, F. Lartaud, Clumped isotopes in modern marine bivalves. *Geochim. Cosmochim. Acta* **316**, 41–58 (2022).

37. E. J. Judd, B. H. Wilkinson, L. C. Ivany, The life and time of clams: Derivation of intra-annual growth rates from high-resolution oxygen isotope profiles. *Palaeogeogr. Palaeoclimatol. Palaeoecol.* **490**, 70–83 (2018).

38. J. M. Eiler, Paleoclimate reconstruction using carbonate clumped isotope thermometry. *Quat. Sci. Rev.* **30**, 3575–3588 (2011).

39. D. A. Stolper, J. M. Eiler, The kinetics of solid-state isotope-exchange reactions for clumped isotopes: A study of inorganic calcites and apatites from natural and experimental samples. *Am. J. Sci.* **315**, 363–411 (2015).

40. J. D. Hemingway, G. A. Henkes, A disordered kinetic model for clumped isotope bond reordering in carbonates. *Earth Planet. Sci. Lett.* **566**, 116962 (2021).

41. T. Y. Wang, R. Hoffmann, S. L. He, Q. H. Zhang, G. B. Li, H. N. Randrianaly, J. Xie, Y. H. Yue, L. Ding, Early Cretaceous climate for the southern Tethyan Ocean: Insights from the geochemical and paleoecological analyses of extinct cephalopods. *Glob. Planet. Change* **229**, 104220 (2023).

42. J. R. Kelson, K. W. Huntington, A. J. Schauer, C. Saenger, A. R. Lechler, Toward a universal carbonate clumped isotope calibration: Diverse synthesis and preparatory methods suggest a single temperature relationship. *Geochim. Cosmochim. Acta* **197**, 104–131 (2017).

43. S. V. Petersen, W. F. Defliese, C. Saenger, M. Daëron, K. W. Huntington, C. M. John, J. R. Kelson, S. M. Bernasconi, A. S. Colman, T. Kluge, G. A. Olack, A. J. Schauer, D. Bajnai, M. Bonifacie, S. F. M. Breitenbach, J. Fiebig, A. B. Fernandez, G. A. Henkes, D. Hodell, A. Katz, S. Kele, K. C. Lohmann, B. H. Passey, M. Y. Peral, D. A. Petrizzo, B. E. Rosenheim, A. Tripati, R. Venturelli, E. D. Young, I. Z. Winkelstern, Effects improved ^{17}O correction on inter-laboratory agreement in clumped isotope calibrations, estimates of mineral-specific offsets, and temperature dependence of acid digestion fractionation. *Geochem. Geophys. Geosyst.* **20**, 3495–3519 (2019).

44. N. Meinicke, S. L. Ho, B. Hannisdal, D. Nürnberg, A. Tripati, R. Schiebel, A. N. Meckler, A robust calibration of the clumped isotopes to temperature relationship for foraminifers. *Geochim. Cosmochim. Acta* **270**, 160–183 (2020).

45. A. N. Meckler, P. F. Sexton, A. M. Piasecki, T. J. Leutert, J. Marquardt, M. Ziegler, T. Agterhuis, L. J. Lourens, J. W. B. Rae, J. Barnet, A. Tripati, S. M. Bernasconi, Cenozoic evolution of deep ocean temperature from clumped isotope thermometry. *Science* **377**, 86–90 (2022).

46. N. T. Anderson, J. R. Kelson, S. Kele, M. Daëron, M. Bonifacie, J. Horita, T. J. Mackey, C. M. John, T. Kluge, P. Petschnig, A. B. Jost, K. W. Huntington, S. M. Bernasconi, K. D. Bergmann, A unified clumped isotope thermometer calibration (0. 5–1,100 °C) using carbonate-based standardization. *Geophys. Res. Lett.* **48**, e2020GL092069 (2021).

47. S. Kele, S. F. M. Breitenbach, E. Capezzuoli, A. N. Meckler, M. Ziegler, I. M. Millan, T. Kluge, J. Deák, K. Hanselmann, C. M. John, H. Yan, Z. H. Liu, S. M. Bernasconi, Temperature dependence of oxygen- and clumped isotope fractionation in carbonates: A study of travertines and tufas in the 6-95 °C temperature range. *Geochim. Cosmochim. Acta* **168**, 172–192 (2015).

48. S. M. Bernasconi, I. A. Müller, K. D. Bergmann, S. F. M. Breitenbach, A. Fernandez, D. A. Hodell, M. Jaggi, A. N. Meckler, I. Millan, M. Ziegler, Reducing uncertainties in carbonate clumped isotope analysis through consistent carbonate-based standardization. *Geochem. Geophys. Geosyst.* **19**, 2895–2914 (2018).

49. M. Peral, M. Daëron, D. Blamart, F. Bassinot, F. Dewilde, N. Smialkowski, G. Isguder, J. Bonnin, F. Jorissen, C. Kissel, E. Michel, N. V. Riveiros, C. Waelbroeck, Updated calibration of the clumped isotope thermometer in planktonic and benthic foraminifera. *Geochim. Cosmochim. Acta* **239**, 1–16 (2018).

50. A. J. Davies, S. Davis, C. M. John, Evidence of taxonomic non-equilibrium effects in the clumped isotope composition of modern cephalopod carbonate. *Chem. Geol.* **578**, 120317 (2021).

51. Y. Guo, W. Deng, G. Wei, X. Chen, X. Liu, X. Wang, L. Lo, G. Cai, T. Zeng, Exploring the temperature dependence of clumped isotopes in modern Porites corals. *J. Geophys. Res. Biogeosci.* **125**, e2019JG005402 (2020).

52. A. J. Davies, W. Guo, M. Bernecker, M. Tagliavento, J. Raddatz, E. Gischler, S. Flögel, J. Fiebig, Dual clumped isotope thermometry of coral carbonate. *Geochim. Cosmochim. Acta* **338**, 66–78 (2022).

53. G. A. Henkes, B. H. Passey, A. D. Wanamaker, E. L. Grossman, W. G. Ambrose, M. L. Carroll, Carbonate clumped isotope compositions of modern marine mollusk and brachiopod shells. *Geochim. Cosmochim. Acta* **106**, 307–325 (2013).

54. D. Bajnai, W. Guo, C. Spötl, T. B. Coplen, K. Methner, N. Löffler, E. Krsnik, E. Gischler, M. Hansen, D. Henkel, G. D. Price, J. Raddatz, D. Scholz, J. Fiebig, Dual clumped isotope thermometry resolves kinetic biases in carbonate formation temperatures. *Nat. Commun.* **11**, 4005 (2020).

55. J. F. Kniest, A. J. Davies, J. Brugger, J. Fiebig, M. Bernecker, J. A. Todd, T. Hickler, S. Voigt, A. Woodland, J. Raddatz, Dual clumped isotopes from Mid-Eocene bivalve shell reveal a hot and summer wet climate of the Paris Basin. *Commun. Earth Environ.* **5**, 330 (2024).

56. K. J. Dennis, J. K. Cochran, N. H. Landman, D. P. Schrag, The climate of the Late Cretaceous: New insights from the application of the carbonate clumped isotope thermometer to Western Interior Seaway macrofossil. *Earth Planet. Sci. Lett.* **362**, 51–65 (2013).

57. Y. Iba, K.-I. Hirauchi, The Early Cretaceous in situ shallow-marine carbonates containing typical Tethyan biota in the Ishido Formation, Kanto Mountains, central Japan. *J. Geol. Soc. Japan* **115**, V–VI (2009).

58. C. V. Ullmann, F. Böhm, R. E. M. Rickaby, U. Wiechert, C. Korte, The Giant Pacific Oyster (*Crassosrea gigas*) as a modern analog for fossil ostreoids: Isotopic (Ca, O, C) and elemental (Mg/Ca, Sr/Ca, Mn/Ca) proxies. *Geochem. Geophy. Geosy.* **14**, 4109–4120 (2013).

59. S. F. M. Breitenbach, M. J. Mleneck-Vautravers, A.-L. Grauel, L. Lo, S. M. Bernasconi, I. A. Müller, J. Rolfe, F. Gázquez, M. Greaves, D. A. Hodell, Coupled Mg/Ca and clumped isotope analyses of foraminifera provide consistent water temperatures. *Geochim. Cosmochim. Acta* **236**, 283–296 (2018).

60. N. J. de Winter, S. Goderis, F. Dehairs, J. W. M. Jagt, R. H. B. Fraaije, S. J. M. Van Malderen, F. Vanhaecke, P. Claeys, Tropical seasonality in the late Campanian (late Cretaceous): Comparison between multiproxy records from three bivalve taxa from Oman. *Palaeogeogr. Palaeoclimatol. Palaeoecol.* **485**, 740–760 (2017).

61. N. J. de Winter, S. Goderis, S. J. M. Van Malderen, M. Sinnesael, S. Vansteenberge, C. Snoeck, J. Belza, F. Vanhaecke, P. Claeys, Subdaily-scale chemical variability in a Torreites sanchezi rudist shell: Implications for rudist paleobiology and the Cretaceous day-night cycle. *Paleoceanography and Paleoclimatology* **35**, e2019PA003723 (2020).

62. L. J. de Nooijer, H. J. SperoErez, J. Bijma, G. J. Reichart, Biomineralization in perforate foraminifera. *Earth-Sci. Rev.* **135**, 48–58 (2014).

63. S. R. Durham, D. P. Gillikin, D. H. Goodwin, G. P. Dietl, Rapid determination of oyster lifespans and growth rates using LA-ICP-MS line scans of shell Mg/Ca ratios. *Palaeogeogr. Palaeoclimatol. Palaeoecol.* **485**, 201–209 (2017).

64. P. Meissner, J. Mutterlose, S. Bodin, Latitudinal temperature trends in the northern hemisphere during the Early Cretaceous (Valanginian-Hauterivian). *Palaeogeogr. Palaeoclimatol. Palaeoecol.* **424**, 17–39 (2015).

65. L. C. Ivany, E. J. Judd, Deciphering temperature seasonality in Earth's ancient oceans. *Annu. Rev. Earth Planet. Sci.* **50**, 123–152 (2022).

66. S. He, L. Ding, Z. Xiong, R. A. Spicer, A. Farnsworth, P. J. Valdes, C. Wang, F. Cai, H. Wang, Y. Sun, D. Zeng, J. Xie, Y. Yue, C. Zhao, P. Song, C. Wu, A distinctive Eocene Asian monsoon and modern biodiversity resulted from the rise of eastern Tibet. *Sci. Bull.* **67**, 2245–2258 (2022).

67. A. Licht, M. van Cappelle, H. A. Abels, J. B. Ladant, J. Trabucho-Alexandre, C. France-Lanord, Y. Donnadieu, J. Vandenberghe, T. Rigaudier, C. Lecuyer, D. Terry Jr., R. Adriaens, A. Boura, Z. Guo, A. N. Soe, J. Quade, G. Dupont-Nivet, J. J. Jaeger, Asian monsoons in a late Eocene greenhouse world. *Nature* **513**, 501–506 (2014).

68. D. R. Gröcke, G. D. Price, S. A. Robinson, E. Y. Baraboshkin, J. Mutterlose, A. H. Ruffell, The Upper Valanginian (Early Cretaceous) positive carbon-isotope event recorded in terrestrial plants. *Earth Planet. Sci. Lett.* **240**, 495–509 (2005).

69. M. E. Jelby, K. K. Śliwińska, M. J. Koevoets, P. Alsen, M. L. Vickers, S. Olaussen, L. Stemmerik, Arctic reappraisal of global carbon-cycle dynamics across the Jurassic–Cretaceous boundary and Valanginian Weissert Event. *Palaeogeogr. Palaeoclimatol. Palaeoecol.* **555**, 109847 (2020).

70. K. Littler, S. A. Robinson, P. R. Bown, A. J. Nederbragt, R. D. Pancost, High sea-surface temperatures during the Early Cretaceous Epoch. *Nat. Geosci.* **4**, 169–172 (2011).

71. G. D. Price, B. H. Passey, Dynamic polar climates in a greenhouse world: Evidence from clumped isotope thermometry of Early Cretaceous belemnites. *Geology* **41**, 923–926 (2013).

72. M. L. Vickers, D. Bajnai, G. D. Price, J. Linckens, J. Fiebig, Southern high latitude warmth during Jurassic-Cretaceous: New evidence from clumped isotope thermometry. *Geology* **47**, 724–728 (2019).

73. K. E. Snell, B. L. Thrasher, J. M. Eiler, P. L. Koch, L. C. Sloan, N. J. Tabor, Hot summers in the Bighorn Basin during the early Paleogene. *Geology* **41**, 55–58 (2013).

74. L. Burgener, E. Hyland, K. W. Huntington, J. R. Kelson, J. O. Sewall, Revisiting the equable climate problem during the Late Cretaceous greenhouse using paleosol carbonate clumped isotope temperatures from the Campanian of the Western Interior Basin, USA. *Palaeogeogr. Palaeoclimatol. Palaeoecol.* **516**, 244–267 (2019).

75. C. J. Poulsen, D. Pollard, T. S. White, General circulation model simulation of the $\delta^{18}O$ content of continental precipitation in the middle Cretaceous: A model-proxy comparison. *Geology* **35**, 199–202 (2007).

76. J.-B. Ladant, Y. Donnadieu, Palaeogeographic regulation of glacial events during the Cretaceous supergreenhouse. *Nat. Commun.* **7**, 12771 (2016).

77. J. Landwehrs, G. Feulner, S. Petri, B. Sames, M. Wagreich, Investigating Mesozoic climate trends and sensitivities with a large ensemble of climate model simulations. *Paleoceanography and Paleoclimatology* **36**, e2020PA004134 (2021).

78. D. J. Lunt, A. Farnsworth, C. Loptson, G. L. Foster, P. Markwick, C. L. O'Brien, R. D. Pancost, S. A. Robinson, N. Wrobel, Palaeogeographic controls on climate and proxy interpretation. *Clim. Past* **12**, 1181–1198 (2016).

79. A. Farnsworth, D. J. Lunt, C. L. O'Brien, G. L. Foster, G. N. Inglis, P. Markwick, R. D. Pancost, S. A. Robinson, Climate sensitivity on geological timescales controlled by nonlinear feedbacks and ocean circulation. *Geophys. Res. Lett.* **46**, 9880–9889 (2019).

80. C. M. Huang, G. J. Retallack, C. S. Wang, Early Cretaceous atmospheric P_{CO_2} levels recorded from pedogenic carbonates in China. *Cretaceous Res.* **33**, 42–49 (2012).

81. N. J. Shackleton, J. P. Kennett, Paleotemperature history of the Cenozoic and the initiation of Antarctic glaciation: Oxygen and carbon isotope analyses in DSDP sites 277, 279, and 281. *Init. Repts. DSDP* **29**, 743–755 (1975).

82. K. Wallmann, Impact of atmospheric CO_2 and galactic cosmic radiation on Phanerozoic climate change and the marine $\delta^{18}O$ record. *Geochem. Geophys. Geosyst.* **5**, doi.org/10.1029/2003GC000683 (2004).

83. H. Wierzbowski, D. Bajnai, U. Wacker, M. A. Rogov, J. Fiebig, E. M. Tesakova, Clumped isotope record of salinity variations in the Subboreal Province at the middle–late Jurassic transition. *Global Planet. Change* **167**, 172–189 (2018).

84. E. Kemper, H.-H. Schmitz, Stellate nodules from the upper Deer Bay Formation (Valanginian) of Arctic Canada. *Geol. Surv. Prof. Pap.* **75**, 109–119 (1975).

85. G. D. Price, E. V. Nunn, Valanginian isotope variation in glendonites and belemnites from Arctic Svalbard: Transient glacial temperatures during the Cretaceous greenhouse. *Geology* **38**, 251–254 (2010).

86. N. F. Alley, S. B. Hore, L. A. Frakes, Glaciations at high-latitude Southern Australia during the Early Cretaceous. *Aust. J. Earth Sci.* **67**, 1045–1095 (2020).

87. S. E. Grasby, G. E. McCune, B. Beauchamp, J. M. Galloway, Lower Cretaceous cold snaps led to widespread glendonite occurrences in the Sverdrup Basin, Canadian High Arctic. *GSA Bull.* **129**, 771–787 (2017).

88. L. A. Frakes, J. E. Francis, A guide to Phanerozoic cold polar climates from high latitude ice-rafting in the Cretaceous. *Nature* **333**, 547–549 (1988).

89. G. D. Price, The evidence and implications of polar ice during the Mesozoic. *Earth Sci. Rev.* **48**, 183–210 (1999).

90. R. M. DeConto, D. Pollard, Rapid Cenozoic glaciation of Antarctica induced by declining atmospheric CO_2. *Nature* **421**, 245–249 (2003).

91. T. Y. Wang, S. L. He, Q. H. Zhang, L. Ding, A. Farnsworth, F. L. Cai, C. Wang, J. Xie, G. B. Li, J. N. Sheng, Y. H. Yue, Ice sheet expansion in the Cretaceous greenhouse world. *Fundam. Res.* **4**, 1586–1593 (2023).

92. M. L. Vickers, S. K. Lengger, S. M. Bernasconi, N. Thibault, B. P. Schultz, A. Fernandez, C. V. Ullmann, P. McCormack, C. J. Bjerrum, J. A. Rasmussen, I. W. Hougård, C. Korte, Cold spells in the Nordic Seas during the early Eocene Greenhouse. *Nat. Commun.* **11**, 4713 (2020).

93. M. Rogov, V. Ershova, O. Vereshchagin, K. Vasileva, K. Mikhailova, A. Krylov, Database of global glendonite and ikaite records throughout the Phanerozoic. *Earth Syst. Sci. Data* **13**, 343–356 (2021).

94. M. L. Vickers, M. Vickers, R. E. M. Rickaby, H. Wu, S. M. Bernasconi, C. V. Ullmann, G. Bohrmann, R. F. Spielhagen, H. Kassens, B. P. Schultz, C. Alwmark, N. Thibault, C. Korte, The ikaite to calcite transformation: Implications for palaeoclimate studies. *Geochim. Cosmochim. Acta* **334**, 201–216 (2022).

95. C. F. Li, J. H. Guo, Z. Y. Chu, L. J. Feng, X. C. Wang, Direct high-precision measurements of the $^{87}Sr/^{86}Sr$ isotope thermal ionization mass spectrometry equipped with 1012 Ω resistors. *Anal. Chem.* **87**, 7426–7432 (2015).

96. J. M. McArthur, R. J. Howarth, G. A. Shields, Y. Zhou, "Chapter 7-Strontium isotope stratigraphy" in *Geologic Time Scale 2020* (Elsevier, 2020).

97. C. M. John, D. Bowen, Community software for challenging isotope analysis: First applications of 'Easotope' to clumped isotopes. *Rapid Commun. Mass Spectrom.* **30**, 2285–2300 (2016).

98. W. A. Brand, S. S. Assonov, T. B. Coplen, Correction for the ^{17}O interference in $\delta^{13}C$ measurements when analyzing CO_2 with stable isotope mass spectrometry (IUPAC Technical Report). *Pure Appl. Chem.* **82**, 1719–1733 (2010).

99. K. W. Huntington, J. M. Eiler, H. P. Affek, W. Guo, M. Bonifacie, L. Y. Yeung, N. Thiagarajan, B. Passey, A. Tripati, M. Daëron, R. Came, Methods and limitations of 'clumped' CO_2 isotope (Δ_{47}) analysis by gas-source isotope ratio mass spectrometry. *J. Mass Spectrom.* **44**, 1318–1329 (2009).

100. K. J. Dennis, H. P. Affek, B. H. Passey, D. P. Schrag, J. M. Eiler, Defining an absolute reference frame for 'clumped' isotope studies of CO_2. *Geochim. Cosmochim. Acta* **75**, 7117–7131 (2011).

101. S. M. Bernasconi, M. Daëron, K. D. Bergmann, M. Bonifacie, A. N. Meckler, H. P. Affek, N. Anderson, D. Bajnai, E. Barkan, E. Beverly, D. Blamart, L. Burgener, D. Calmels, C. Chaduteau, M. Clog, B. Davidheiser-Kroll, A. Davies, F. Dux, J. Eiler, B. Elliott, A. C. Fetrow, J. Fiebig, S. Goldberg, M. Hermoso, K. W. Huntington, E. Hyland, M. Ingalls, M. Jaggi, C. M. John, A. B. Jost, S. Katz, J. Kelson, T. Kluge, I. J. Kocken, A. Laskar, T. J. Leutert, D. Liang, J. Lucarelli, T. J. Mackey, X. Mangenot, N. Meinicke, S. E. Modestou, I. A. Müller, S. Murray, A. Neary, N. Packard, B. H. Passey, E. Pelletier, S. Petersen, A. Piasecki, A. Schauer, K. E. Snell, P. K. Swart, A. Tripati, D. Upadhyay, T. Vennemann, I. Winkelstern, D. Yarian, N. Yoshida, N. Zhang, M. Ziegler, InterCarb: A community effort to improve interlaboratory standardization of the carbonate clumped isotope thermometer using carbonate standards. *Geochem. Geophys. Geosyst.* **22**, e2020GC009588 (2021).

102. B. Chang, C. Li, D. Liu, I. Foster, A. Tripati, M. K. Lloyd, I. Maradiaga, G. M. Luo, Z. H. An, Z. B. She, S. C. Xie, J. N. Tong, J. H. Huang, T. J. Algeo, T. W. Lyons, A. Immenhauser, Massive formation of early diagenetic dolomite in the Ediacaran ocean: Constraints on the "dolomite problem". *Proc. Natl. Acad. Sci. U.S.A.* **117**, 14005–14014 (2020).

103. M. M. Jones, S. V. Petersen, A. N. Curley, A tropically hot mid-Cretaceous North American Western Interior Seaway. *Geology* **50**, 954–958 (2022).

104. J. Z. Zhang, S. V. Petersen, Clumped and oxygen isotope sclerochronology methods tested in the bivalve *Lucina pensylvanica*. *Chem. Geol.* **620**, 121346 (2023).

105. M. L. Vickers, A. Fernandez, S. P. Hesselbo, G. D. Price, S. M. Bernasconi, S. Lode, C. V. Ullmann, N. Thibault, I. W. Hougaard, C. Korte, Unravelling Middle to Late Jurassic

palaeoceanographic and palaeoclimatic signals in the Hebrides Basin using belemnite clumped isotope thermometry. *Earth Planet. Sci. Lett.* **546**, 116401 (2020).

106. A. Fernandez, I. A. Müller, L. Rodríguez-Sanz, J. van Dijk, N. Looser, S. M. Bernasconi, A reassessment of the precision of carbonate clumped isotope measurements: Implications for calibrations and paleoclimate reconstructions. *Geochem. Geophy. Geosy.* **18**, 4375–4386 (2017).

107. P. J. Valdes, E. Armstrong, M. P. S. Badger, C. D. Bradshaw, F. Bragg, M. Crucifix, T. Davies-Barnard, J. J. Day, A. Farnsworth, C. Gordon, P. O. Hopcroft, A. T. Kennedy, N. S. Lord, D. J. Lunt, A. Marzocchi, L. M. Parry, V. Pope, W. H. G. Roberts, E. J. Stone, G. J. L. Tourte, J. H. T. Williams, The BRIDGE HadCM3 family of climate models: HadCM3@ Bristol v1.0. *Geosci. Model Dev.* **10**, 3715–3743 (2017).

108. P. M. Cox, R. A. Betts, C. B. Bunton, R. L. H. Essery, P. R. Rowntree, J. Smith, The impact of new land surface physics on the GCM simulation of climate and climate sensitivity. *Clim. Dyn.* **15**, 183–203 (1999).

109. J. Tindall, R. Flecker, P. Valdes, D. N. Schmidt, P. Markwick, J. Harris, Modelling the oxygen isotope distribution of ancient seawater using a coupled ocean-atmosphere GCM: Implications for reconstructing early Eocene climate. *Earth Planet. Sci. Lett.* **292**, 265–273 (2010).

110. A. Farnsworth, P. J. Valdes, L. Ding, R. A. Spicer, S.-H. Li, T. Su, S. Li, C. R. Witkowski, Z. Xiong, Limits of oxygen isotope palaeoaltimetry in Tibet. *Earth Planet. Sci. Lett.* **606**, 118040 (2023).

111. J. C. Tindall, P. J. Valdes, L. C. Sime, Stable water isotopes in HadCM3: Isotopic signature of EI Niño-Southern Oscillation and the tropical amount effect. *J. Geophys. Res.* **114**, doi.org/10.1029/2008JD010825 (2009).

112. P. J. Valdes, C. R. Scotese, D. J. Lunt, Deep ocean temperatures through time. *Clim. Past* **17**, 1483–1506 (2021).

113. G. L. Foster, D. L. Royer, D. J. Lunt, Future climate forcing potentially without precedent in the last 420 million years. *Nat. Commun.* **8**, 14845 (2017).

114. L. D. Ashwal, R. Tucker, Geology of Madagascar: A brief outline. *Gondw. Res.* **2**, 335–339 (1999).

115. R. D. Tucker, L. D. Ashwall, M. J. Handke, M. A. Hamilton, M. Le Grange, R. A. Rambeloson, U–Pb geochronology and isotope geochemistry of the Archean and Proterozoic rocks of north-central Madagascar. *J. Geol.* **107**, 135–153 (1999).

116. M. Geiger, D. N. Clark, W. Mette, Reappraisal of the timing of the breakup of Gondwana based on sedimentological and seismic evidence from the Morondava Basin, Madagascar. *J. Afr. Earth Sci.* **38**, 363–381 (2004).

117. J. V. Lalaharisaina, N. J. Ferrand, Cretaceous may hold promise in Majunga Basin, Madagascar. *Oil Gas J.* **1**, 54–58 (1994).

118. M. F. Coffin, P. D. Rabinowitz, "The Mesozoic East African and Madagascan conjugate continental margins: Stratigraphy and tectonics" in *Geology and Geophysics of Continental Margins*, J. S. Watkins, Z. Feng, K. J. McMillen, Eds. (American Association of Petroleum Geologists, 1992), pp. 207–240.

119. J. Boast, A. E. M. Nairn, "Chapter 14—An outline of the geology of Madagascar" in *The Ocean Basins and Margins-Volume 6-The Indian Ocean*, A. E. M. Nairn, F. G. Stehli, Eds. (Plenum Press, 1982), pp. 649–696.

120. Y. Razafindrazaka, T. Randriamananjara, A. Piqué, C. Thouin, E. Laville, J. Malod, J.-P. Réhault, Late Paleozoic and Mesozoic extension and sedimentation in the Majunga Basin (northwestern Madagascar). *J. Afr. Earth Sci.* **28**, 949–959 (1999).

121. G. A. Henkes, B. H. Passey, E. L. Grossman, B. J. Shenton, A. Pérez-Huerta, T. E. Yancey, Temperature limits for preservation of primary calcite clumped isotope paleotemperatures. *Geochim. Cosmochim. Acta* **139**, 362–382 (2014).

122. A. Fernandez, C. Korte, C. V. Ullmann, N. Looser, S. Wohlwend, M. S. Bernasconi, Reconstructing the magnitude of Early Toarcian (Jurassic) warming using the recorded clumped isotope compositions of belemnites. *Geochim. Cosmochim. Acta* **293**, 308–327 (2021).

123. M. R. Carriker, R. E. Palmer, Ultrastructural morphogenesis of prodissoconch and early dissoconch valves of the Oyster *Crassostrea virginica*. *Proc. Natl. Shellfish. Ass.* **69**, 103–128 (1979).

124. C. V. Ullmann, U. Wiechert, C. Korte, Oxygen isotope fluctuations in a modern North Sea oyster (*Crassostrea gigas*) compared with annual variations in seawater temperature: Implications for palaeoclimate studies. *Chem. Geol.* **277**, 160–166 (2010).

125. K. Chinzei, A. Seilacher, Remote biomineralization I: Fill skeletons in vesicular oyster shells. *Neues Jahrb. Geol. Paläontol.* **190**, 349–361 (1993).

126. E. L. Grossman, "Applying oxygen isotope paleothermometry in deep time" in *Reconstructing Earth's Deep-Time Climate: State of the Art in 2012*, L. Ivany, B. Huber, Eds. (The Paleontological Society Papers, Paleontological Society Short Course, 3 November 2021, 2012), pp. 39–67, vol. 18.

127. C. V. Ullmann, C. Korte, C. Korte, Diagenetic alteration in low-Mg calcite from macrofossils: A review. *Geology* **59**, 3–20 (2015).

128. M. I. Benito, M. Reolid, Belemnite taphonomy (Upper Jurassic, Western Tethys) part II: Fossil–diagenetic analysis including combined petrographic and geochemical techniques. *Palaeogeogr. Palaeoclimatol. Palaeoecol.* **358–360**, 89–108 (2012).

129. N. F. Goodkin, K. A. Hughen, A. L. Cohen, A multicoral calibration method to approximate a universal equation relating Sr/Ca and growth rate to sea surface temperature. *Paleoceanography* **22**, doi.org/10.1029/2006PA001312 (2007).

130. J. R. Dodd, Environmental control of strontium and magnesium in Mytilus. *Geochim. Cosmochim. Acta* **29**, 385–398 (1965).

131. C. S. Füllenbach, B. R. Schöne, K. Shirai, N. Takahata, A. Ishida, Y. Sano, Minute co-variations of Sr/Ca ratios and microstructures in the aragonitic shell of Cerastoderma edule (Bivalvia)—Are geochemical variations at the ultra-scale masking potential environmental signals? *Geochim. Cosmochim. Acta* **205**, 256–271 (2017).

132. P. S. Freitas, L. J. Clarke, H. Kennedy, C. A. Richardson, F. Abrantes, Environmental and biological controls on elemental (Mg/Ca, Sr/Ca and Mn/Ca) ratios in shells of the king scallop *Pecten maximus*. *Geochim. Cosmochim. Acta* **70**, 5119–5133 (2006).

133. P. S. Freitas, L. J. Clarke, H. Kennedy, C. A. Richardson, Manganese in the shell of the bivalve *Mytilus edulis*: Seawater Mn or physiological control? *Geochim. Cosmochim. Acta* **194**, 266–278 (2016).

134. W. S. Moore, M. Beck, T. Riedel, M. Rutgers van der Loeff, O. Dellwig, T. J. Shaw, B. Schnetger, H. J. Brumsack, Radium-based pore water fluxes of silica, alkalinity, manganese, DOC, and uranium: A decade of studies in the German Wadden Sea. *Geochim. Cosmochim. Acta* **75**, 6535–6555 (2011).

135. M. Beck, O. Dellwig, B. Schnetger, T. Riedel, H. J. Brumsack, Manganese dynamics in tidal basins of the Wadden Sea: Spatial/seasonal patterns and budget estimates. *Mar. Chem.* **225**, 103847 (2020).

136. M. Beck, O. Dellwig, B. Schnetger, H. J. Brumsack, Cycling of trace metals (Mn, Fe, Mo, U, V, Cr) in deep pore waters of intertidal flat sediments. *Geochim. Cosmochim. Acta* **72**, 2822–2840 (2008).

137. E. V. Nunn, G. D. Price, Late Jurassic (Kimmeridgian–Tithonian) stable isotopes ($\delta^{18}O$, $\delta^{13}C$) and Mg/Ca ratios: New palaeoclimate data from Helmsdale, northeast Scotland. *Palaeogeogr. Palaeoclimatol. Palaeoecol.* **292**, 325–335 (2010).

138. G. D. Price, A. H. Ruffell, C. E. Jones, R. M. Kalin, J. Mutterlose, Isotopic evidence for temperature variation during the Early Cretaceous. *J. Geol. Soc.* **157**, 335–343 (2000).

139. B. van de Schootbrugge, K. Follmi, L. G. Bulot, S. J. Burns, Paleoceanographic changes during the early Cretaceous (Valanginian–Hauterivian): Evidence from oxygen and carbon stable isotopes. *Earth Planet. Sci. Lett.* **181**, 15–31 (2000).

140. G. D. Price, N. M. M. Janssen, M. Martinez, M. Company, J. H. Vandevelde, S. T. Grimes, A high-resolution belemnite geochemical analysis of Early Cretaceous (Valanginian-Hauterivian) environmental and climatic perturbations. *Geochem. Geophys. Geosyst.* **19**, 3832–3843 (2018).

141. S. Finnegan, K. Bergmann, J. M. Eiler, D. S. Jones, D. A. Fike, I. Eisenman, N. C. Hughes, A. K. Tripati, W. W. Fischer, The magnitude and duration of Late Ordovician–Early Silurian glaciation. *Science* **331**, 903–906 (2011).

142. J. Mutterlose, S. Bodin, L. Fähnrich, Strontium-isotope stratigraphy of the Early Cretaceous (Valanginian–Barremian): Implications for Boreal–Tethys correlation and paleoclimate. *Cretac. Res.* **50**, 252–263 (2014).
